# A Mechanistic Weather-Driven Model for *Ascochyta rabiei* Infection and Disease Development in Chickpea

**DOI:** 10.3390/plants10030464

**Published:** 2021-03-01

**Authors:** Irene Salotti, Vittorio Rossi

**Affiliations:** Department of Sustainable Crop Production (DI.PRO.VES.), Università Cattolica del Sacro Cuore, Via E. Parmense 84, 29122 Piacenza, Italy; irene.salotti1@unicatt.it

**Keywords:** epidemiology, disease modelling, *Ascochyta rabiei*, model evaluation

## Abstract

Ascochyta blight caused by *Ascochyta rabiei* is an important disease of chickpea. By using systems analysis, we retrieved and analyzed the published information on *A. rabiei* to develop a mechanistic, weather-driven model for the prediction of Ascochyta blight epidemics. The ability of the model to predict primary infections was evaluated using published data obtained from trials conducted in Washington (USA) in 2004 and 2005, Israel in 1996 and 1998, and Spain from 1988 to 1992. The model showed good accuracy and specificity in predicting primary infections. The probability of correctly predicting infections was 0.838 and the probability that there was no infection when not predicted was 0.776. The model’s ability to predict disease progress during the growing season was also evaluated by using data collected in Australia from 1996 to 1998 and in Southern Italy in 2019; a high concordance correlation coefficient (CCC = 0.947) between predicted and observed data was obtained, with an average distance between real and fitted data of root mean square error (RMSE) = 0.103, indicating that the model was reliable, accurate, and robust in predicting seasonal dynamics of Ascochyta blight epidemics. The model could help growers schedule fungicide treatments to control Ascochyta blight on chickpea.

## 1. Introduction

Chickpea (*Cicer arietinum* L.) is the most important pulse crop in the world after bean (*Phaseolus vulgaris* L.) and pea (*Pisum sativum* L.) [1]. It is a major source of high-quality protein in human diets and provides high-quality crop residues for animal feed [2]. Furthermore, its nitrogen fixation capability helps maintain soil fertility and cropping system sustainability in cereal-legume rotations [2]. 

Ascochyta blight, caused by the fungus *Ascochyta rabiei* (Pass.) Labrousse [syn. *Phoma rabiei* (Pass.) Khune et Kapoor), teleomorph *Didymella rabiei* (Kovachevski) vs. Arx (syn. *Mycosphaerella rabiei* Kovachevski)], is probably the most serious disease of chickpea worldwide. In 1996, the disease was reported from 35 countries across five continents [3], and a continuous spread into new areas was recorded in the following years [4,5,6,7].

The fungus affects all aboveground parts of the plant causing necrotic lesions on leaves, petioles, stems, pods, and seeds [8], which result in both yield and quality losses [9,10,11]. Yield losses can occasionally reach 100% on susceptible cultivars under favorable environmental conditions [11]. The regular seasonal occurrences of Ascochyta blight epidemics suggest that the fungus has efficient mechanisms for overwintering from one season to the next. The main sources of primary inoculum are infected seeds that cause seedling infection [12,13] and air-borne ascospores produced in pseudothecia formed on chickpea infected debris [14,15]. During the growing season, secondary spread of the disease is driven by splash-borne pycnidiospores (conidia) produced in pycnidia that develop on Ascochyta lesions [11].

Cultural practices may contribute to the control of Ascochyta blight; crop rotation and burial of residue by tillage can reduce the inoculum [16]. The breakdown of infested chickpea residues and the loss of pathogen viability are greater at soil depths >5 cm than in the soil surface [16,17]. Moreover, conidia and ascospores produced on buried residues are not available for either wind or splash dispersal [16]. The cultivation of non-host crops between crops of chickpea and the use of blight-free seeds or fungicide-treated seeds reduces inoculum pressure [16,18]. The exploitation of host-plant resistance is also a management option [2,19,20]. Currently available resistant cultivars, however, show adequate levels of resistance only at the seedling stage, i.e., older plants are susceptible [21,22,23]. 

Management of Ascochyta blight relies strongly on fungicides [2,18,24]; even for cultivars with resistance to Ascochyta blight, one or two fungicide sprays are necessary to achieve disease control [25,26,27]. With susceptible cultivars, up to 12 foliar fungicide sprays are applied for season-long protection [28]. In Victoria, Australia, for example, protective applications are performed every 12–15 days from the seedling stage, and 8–12 spays are typically applied by the end of the chickpea growing season [29]. Although such regimes provide effective Ascochyta blight control, repeated fungicide applications are often uneconomical, especially in areas where chickpea yields are low [2], and may cause negative environmental impacts [30]. The timing of applications must also be considered. Disease control is higher when sprays prevent primary infections by airborne ascospores [31], and a delay of the first application until the late seedling or early flowering stage can lead to poor disease control and yield losses [32]. Therefore, a model for Ascochyta blight should help growers schedule fungicides sprays and thereby control the disease.

Simple empirical models for Ascochyta blight have been developed [33,34,35,36]. Empirical models for pseudothecial maturation have also been conceptualized because of the importance of preventing primary infections [9,31]. However, these models have some limitations: most importantly, they fail to consider either the complexity of the *A. rabiei* life cycle or the susceptibility of the host at different growth stages. As a result, the existing models provide inaccurate estimates of infection risk [9,31,33].

Mechanistic, weather-driven models have been shown to be more accurate and robust than empirical ones [37,38], and can be developed both conceptually and mathematically by using systems analysis [39] and published data [40,41]. 

The overall goal of the current research was to develop a mechanistic model of Ascochyta blight of chickpea. To achieve this goal, we retrieved the relevant information via a systematic literature search and used this information to develop a conceptual model of the *A. rabiei* life cycle based on systems analysis. After using published data to develop mathematical equations that describe the system both quantitatively and dynamically, we finally evaluated the capability of the model to represent the real system.

## 2. Results

### 2.1. Literature Search

A total of 146 papers were obtained by the literature search; among these, 77 papers were selected based on their titles and abstracts, and 5 papers were added based on the listed references. As a result, a total of 82 papers were considered in this study.

### 2.2. Systems Analysis of the A. rabiei Life Cycle

**Figure 1:**
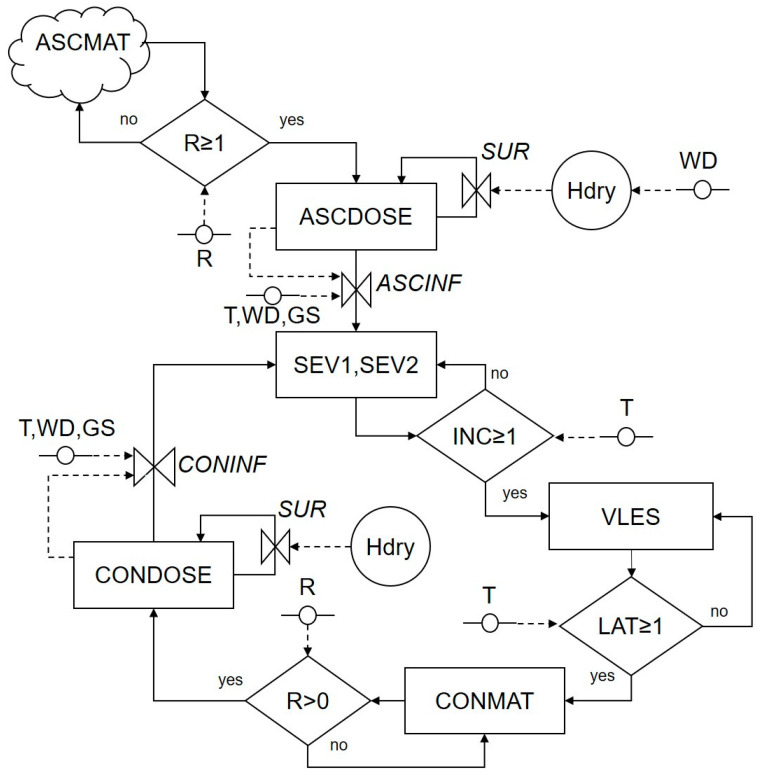
Relational diagram of the life cycle of *Ascochyta rabiei*. Legend: parameters (R, T, WD, GS); boxes are state variables; solid arrows represent flux and direction of states; dotted arrows represent flux and direction of information; circles symbolize intermediate variables; diamonds are switches; “bow ties” are valves in flux (rates) (see Table 1 for acronym explanations).

The relational diagram of the model is shown in Figure 1; variables, switches, and rates of the relational diagram are described in Table 1. The first state variable of the model consists of the mature ascospores in overwintered pseudothecia on chickpea debris (named ASCMAT). Spring rains (R) trigger the release of mature ascospores into the air and their deposition on chickpea plants surfaces; the ascospores that land on these plant surfaces represent the second state variable of the model (ASCDOSE). These ascospores cause infections on leaves, stems, petioles, and pods under favorable conditions through an infection rate (*ASCINF*), or they survive under unfavorable conditions at a survival rate (*SUR*). At the end of an incubation period (INC), Ascochyta blight infections become visible as necrotic lesions (i.e., the fourth state variable VLES; Table 1). At the end of a latency period (LAT), pycnidia bearing mature conidia are produced in lesions (i.e., the fifth state variable CONMAT). Rain splashes disperse the conidia that are then deposited on plant tissues (i.e., the sixth state variable CONDOSE); these conidia either cause secondary infections that enter into infection sites through an infection rate (*CONINF*) or they survive at a survival rate (*SUR*).

The flow from one stage to the following stage is regulated by rates (valves) and switches (diamonds) that are influenced by external variables and auxiliary variables, i.e., by weather variables (temperature, T in °C; wetness duration, WD in hours; and rainfall, R in mm) or by chickpea plant growth stage (GS) (vegetative growth, flowering, and pod formation).

The model makes the following assumptions: (i) the carrying capacity of plant tissues is not a limiting factor so that plant growth, senescence and lesion expansion do not affect the establishment of new infections; (ii) an infection period is a wet period initiated by a rain event that scrubs ascospores from the air or causes the splash-dispersal of conidia; (iii) as a consequence of (ii), wet periods due to dew deposition do not cause infection because there are no ascospores or conidia on the plant surface. Some of these assumptions are discussed later.

The model has a time step of 1 h to better account for the effect of fluctuating temperature and humidity conditions during the day, as well as the influence of interruptions in leaf wetness [38,42].

### 2.3. Model Description

The model has three main compartments: (i) primary infections caused by ascospores produced within overwintered pseudothecia and released into the air following spring rainfalls; (ii) lesion appearance and production of pycnidia carrying mature conidia; and (iii) secondary infections caused by the conidia released from pycnidia on lesions. The model begins to run at the emergence of chickpea plants (BBCH 09) and has a time step of 1 h.

#### 2.3.1. Assumptions for the Primary Inoculum

Primary inoculum sources for *A. rabiei* are infected seeds [12,13] and infested chickpea debris from the previous growing season from which conidia produced by pycnidia and/or ascospores produced by pseudothecia are splash- and air-dispersed to plants, respectively [14,15]. Because the model assumes that farmers plant *A. rabiei*-free seeds and rotate the crops so that no chickpea crop debris is present in the field, air-dispersed ascospores from pseudothecia in the infested chickpea debris that overwintered in neighboring fields are the only relevant source of primary inoculum [9,15,31]. 

The model compartment concerning primary inoculum begins at the emergence of chickpea plants and ends at mid-June [14,15]. In this period, the model assumes that ascospores are present in the chickpea-growing area, are airborne, and are deposited on plant surfaces when a rain event occurs. Because no data were found in the literature on the deposition of *A. rabiei* ascospores, the model assumes that deposition occurs whenever rain exceeds 1 mm h^−1^, as is the case for ascospores of *Venturia inaequalis*, the apple scab fungus [43]; *V. inaequalis* and *A. rabiei* belong to the same taxonomic group (order Pleosporales), and their ascospores are similar in shape and size, measuring 11–15 × 5–7 µm [44], and 12–22 × 5–7 µm [45], respectively. The ascospore dose that is deposited on the crop is not quantified and is assumed to be constant during the primary inoculum season. Possible implications of the assumptions regarding the primary inoculum are considered in the Discussion.

#### 2.3.2. Primary Infections

The model begins a primary infection simulation whenever R ≥ 1 mm h^−1^, and assumes that ASCDOSE = 1. There are as many primary infections as there are rains between plant emergence and mid-June, and the model assumes that ASCDOSE = 1 for each of them. Therefore, the further development of each infection process is calculated as a proportion of this ascospore dose, in a 0 to 1 scale.

Ascospores on the plant surface cause infection according to an infection rate (*ASCINF*), which depends on T, WD, and the chickpea GS, and is calculated by fitting the data of Trapero-Casas and Kaiser [46]. The temperature-dependent equation for infection rate is formulated as a Bete equation [47] in the following form:

*ASCINF*(T) = (4.929 (Teq^1.360^) (1 − Teq))^4.663^ (1)

where Teq = equivalent of temperature, calculated as Teq = (T-Tmin)/(Tmax-Tmin), with Tmin = minimum temperature for infection by ascospores (0 °C) and Tmax = maximum temperature for infection by ascospores (35 °C) [46]; when T > Tmax or T < Tmin, no infection occurs; estimates and standard errors of equation parameters were 4.929 ± 0.200, 1.360 ± 0.068, and 4.663 ± 0.830, with R^2^ = 0.971 (see Supplementary material).

The effect of wetness duration on infection rate is calculated as follows:

*ASCINF*(WD) = 0.021 WD − 0.009 (2)

where WD = cumulative number of hours with leaf wetness. Equation (2) was developed and parametrized by fitting the data from Trapero-Casas and Kaiser [46]; estimates and standard errors of equation parameters were 0.021 ± 0.001 and 0.009 ± 0.020, with R^2^= 0.995 (see Supplementary material).

An infection period begins on the first wet hour following R ≥ 1, ends when a dry period occurs, and continues if wetness is restored. During a dry period that interrupts the leaf wetness period, the model considers ASCINF(WD) = 0 and calculates the survival rate of ascospores as follows:

*SUR* = 1 − 0.017 Hdry (3)

where *SUR* = survival rate of ascospores, which ranges from 1 (all ascospores survive) to 0 (no ascospores survive); and Hdry = number of hours with no leaf wetness. Equation (3) was developed and parametrized by fitting the data from Trapero-Casas and Kaiser [46]; the estimate of the equation parameter and its standard error were 0.017 ± 0.001, with R^2^ = 0.937 (see Supplementary material).

The effect of crop growth stage on the infection rate is accounted for by an age-related correction factor (GS), which considers that the susceptibility of chickpea plants to Ascochyta blight differs among growth stages. Values of GS were derived from Sharma et al. [21], Basandrai et al. [22], and Chongo and Gossen [23], as reported in Table 2.

In each i^th^ hour, the infection rate of ascospores is therefore calculated as follows:

*ASCINF* = *ASCINF(T)ASCINF’(WD)* GS (4)

where *ASCINF* ranges from 0 (there is no infection) to 1 (all ascospores cause infection); and *ASCINF’* is the first-order derivative of Equation (2).

The primary infection severity, SEV1 (with 0 ≤ SEV1 ≤ 1), is finally calculated in each i^th^ hour as follows:

SEV1 = ASCDOSE *ASCINF* (5)

#### 2.3.3. Lesion Appearance and Production of Secondary Inoculum

Ascochyta blight symptoms consist of brown lesions that are circular to elongate and that have many black pycnidia arranged in concentric rings. Lesion appearance (at the end of the incubation period) and subsequent production of pycnidia (at the end of the latency period) are mainly regulated by temperature. In each i^th^ hour, the model calculates the hourly progress of both incubation (INC) and latency (LAT) by using the equations of Magarey et al. [48] in the following forms:

INC = f(T) / INCmin(6)

LAT = f(T) / LATmin(7)

where INCmin and LATmin are the shortest duration of incubation and latency, respectively, at Topt; and f(T) is the equation accounting for the influence of temperature, calculated as follows:

f(T) = (T − Tmin) / (Topt − Tmin) ((Tmax − T) / (Tmax − Topt))^(Tmax − Topt) / (Topt − Tmin)^ (8)

where Tmin = minimum temperature for incubation or latency progress; Topt = optimum temperature for incubation or latency progress; Tmax = maximum temperature for incubation or latency progress; when T < Tmin or T > Tmax, f(T) = 0, and incubation or latency does not proceed. The best fit for incubation was obtained with INCmin = 150 h, Tmin = 2 °C, Topt = 21 °C, and Tmax = 34 °C. The best fit for latency was obtained with LATmin = 168, Tmin = 2 °C, Topt = 21 °C, and Tmax = 34 °C.

Data for fitting Equation (6) were derived from Basandrai et al. [22], Trapero-Casas and Kaiser [49], and Chauhan and Sinha [50], and the fit had R^2^ = 0.887; for equation (7), data were derived from Trapero-Casas and Kaiser [49], with R^2^ = 0.945 (see Supplementary material).

The model accumulates the hourly progress of incubation and latency beginning with the hour when a cohort of ascospores or conidia has established infection; when the sum of hourly progress is ≥1, lesion appearance and pycnidia formation are predicted to occur.

#### 2.3.4. Assumptions for Secondary Inoculum

Conidia (pycnidiospores) produced by pycnidia on lesions are responsible for the secondary spread of the disease through rain splashes [11]. The model assumes that fertile Ascochyta blight lesions (i.e., lesions that overcame the latency period) continue to produce conidia for the entire epidemic, so that mature conidia are always present during the epidemic. There are no data concerning the dynamics of pycnidia and conidia production over time on *A. rabiei* lesions; however, the model assumption is supported by studies conducted with other pathogens that form pycnidia, including *Septoria nodorum* [51] and *Septoria tritici* [52] on wheat, *Guignardia bidwellii* on grapevines [53], *Botryosphaeria dothidea* on pistachio [54], and *Diplodia natalensis* on citrus [55]. Based on these studies, the model considers that, during the Ascochyta blight epidemic, repeated infections occur and numerous mature pycnidia develop on lesions and produce conidia, which are dispersed by rain splashes and cause new infections under suitable environmental conditions. At the end of the latency period, these lesions produce new pycnidia and conidia. At the same time, additional conidia are produced in older fruiting bodies, which undergo several sporulation cycles. In dry periods, conidia may survive within the fruiting bodies for a long time and may be dispersed by future rain events.

The model then assumes that conidia are dispersed by any rain, irrespective of rain amount. As before, this assumption is supported by studies for different species belonging to the same class (Dothideomycetes) or order (Pleosporales) as *A. rabiei*, i.e., *Leptosphaeria maculans* [56], *Septoria nodorum* [57], and *Botryosphaeria dothidea* [58]. The conidia of these species are extruded from pycnidia in mucilage and are dispersed by the first falling raindrops. As was the case for primary inoculum, the dose of conidia that are deposited on the crop with rain splashes is not quantified and is then kept constant. Possible implications of the assumptions regarding the secondary inoculum are considered in the Discussion.

#### 2.3.5. Secondary Infections

The model begins a secondary infection simulation whenever there are mature conidia (CONMAT) and R ≥ 0 mm h^−1^, and it maintains CONDOSE = 1. Therefore, the development of the infection is calculated as a proportion of this dose of conidia.

Conidia develop infections depending on temperature, wetness duration, and the chickpea growth stage, and the infection rate (CONINF) is calculated using Equations (9)–(11):

*CONINF*(T) = (5.200 (Teq ^1.560^) (1 − Teq)) ^1.057^ (9)

where Teq is as before, with Tmin = 0 °C and Tmax = 35 °C [55], and estimates and standard errors of equation parameters are 5.200 ± 0.386, 1.560 ± 0.124, and 1.057 ± 0.185. The equation was developed and parametrized using the data of Trapero-Casas and Kaiser [46,49], Khan [59], and Weltzien and Kaack [60], with R^2^ = 0.978 (see Supplementary material).

*CONINF*(WD) = 1 − 1.0 exp (−0.034 WD) (10)

where WD is as before, and estimates and standard errors of equation parameters are 1.0 ± 0.042 and 0.034 ± 0.004. The equation was developed and parameterized by fitting the data of Trapero-Casas and Kaiser [46,49], Armstrong-Cho et al. [61], Khan [59], and Jhorar et al. [62], with R^2^ = 0.944 (see Supplementary material).

The survival rate (*SUR*) of conidia during dry periods is calculated as for ascospores (i.e., Equation (3)).

Finally,

*CONINF* = *CONINF(T) CONINF’(WD)* GS (11)

where *CONINF* ranges from 0 (there is no infection) to 1 (all conidia cause infection); *CONINF’* is the first-order derivative of Equation (10); and GS is as in Table 2.

The severity of the secondary infection, SEV2 (with 0≤ SEV2 ≤1), is calculated in each i^th^ hour as follows:

SEV2 = CONDOSE *CONINF* (12)

### 2.4. Model Validation

#### 2.4.1. Validation of Primary Infections

Model predictions concerning the occurrence of infection (P +) or no infection (P−) and observation of Ascochyta blight appearance (O +) or no appearance (O−) on chickpea bait plants for each year and location are summarized in Table 3. Altogether, 232 cases (groups of bait plants) were considered; 128 of them showed infection and 104 did not. The Bayesian analysis showed that true positive proportion (TPP, i.e., sensitivity) = 0.77, with 98 of 128 real infections correctly predicted by the model, and that true negative proportion (TNP, i.e., specificity) = 0.82, with 85 of 104 cases with no infection correctly predicted (Table 4). In 30 cases, the model failed to predict a real infection resulting in false negative proportion (FNP) = 0.23. In 19 cases, the model predicted infections that did not occur, resulting in false positive proportion (FPP) = 0.18.

Overall model accuracy was 0.79, and the Youden index was J = TPP – FPP = 0.58 (Table 4). The effectiveness of the model as a predictor was also expressed using likelihood ratios: the likelihood ratio of a positive prediction was likelihood ratio LR( + ) = TPP / FPP = 4.19, while the likelihood ratio of a negative prediction was LR(–) = FNP / TNP = 0.29. A large LR( + ) value (larger than 1) and a small LR(–) value (close to 0) indicate that the posterior probabilities are greater than the prior probabilities, meaning that the model provides useful information about the chance of an infection to occur or not to occur. The prior probability of an infection to occur was P(O + ) = 128 / 232 = 0.55, and not to occur was P(O−) = 104 / 232 = 0.45 (Table 4), while the posterior probabilities that there was an infection when predicted by the model was *P*(P + O + ) = 0.838 and that there was no infection when not predicted was *P*(P − O −) = 0.776.

**Figure 2:**
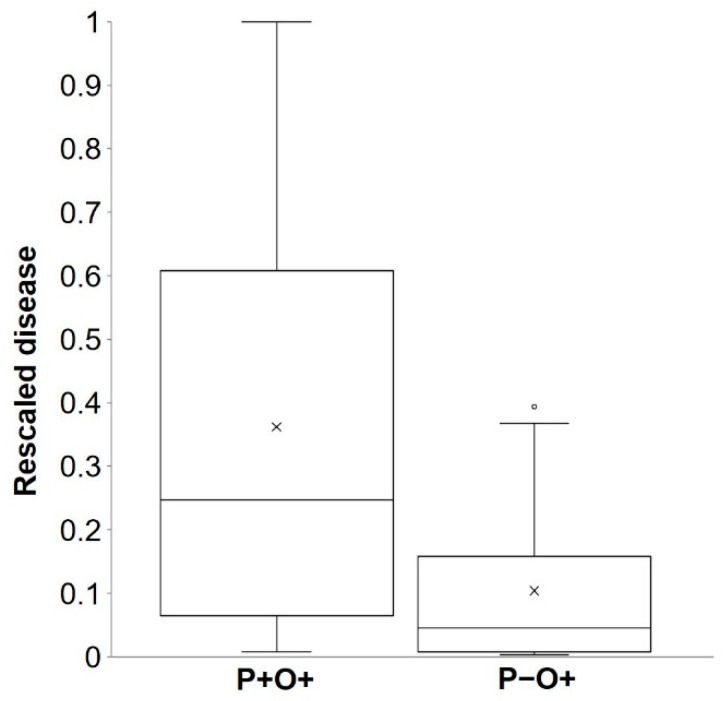
Box plot for rescaled primary infection values (i.e., rescaled relative to the maximum for each year and location) recorded on bait plants for true positive cases (P + O +; left) and false negative cases (P−O+; right) predicted by the model. Observed values were from the 11 experiments listed in Table 3. Boxes include 50% of the data, the horizontal line is the median, the black cross represents the average, whiskers extend to minimum and maximum values, and the empty dot represents an outlier.

**Figure 3:**
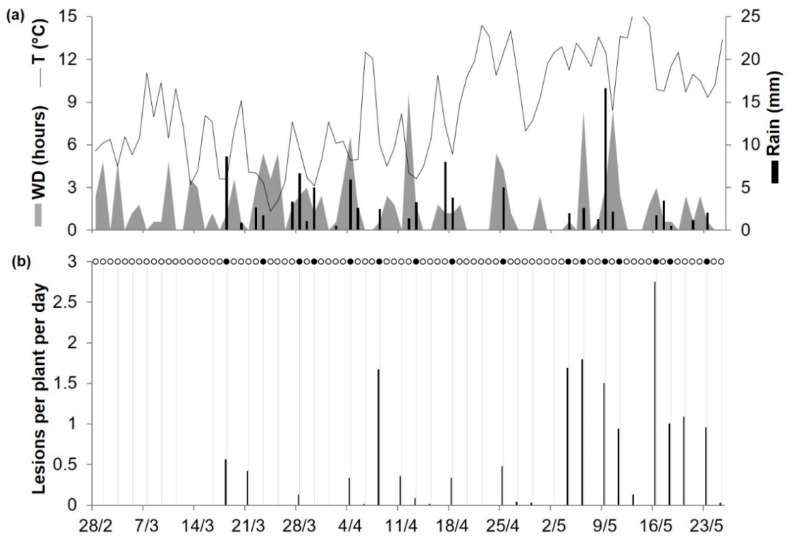
Predicted primary infections and observed primary Ascochyta blight lesions on bait plants (cv. Burpee) exposed to chickpea debris affected by *A. rabiei* at Washington State University’s Spillman Research Farm, US, in 2005. (**a**) Weather variables: air temperature (T, °C, solid line), rainfall (Rain, mm, black bars), and leaf wetness duration (WD, hours, grey area). (**b**) Black bars represent the number of lesions per plant per day observed at the end of the exposure period; grey vertical lines divide different bait plant exposure periods; black points represent days on which infection was predicted by the model; and empty points represent days on which the model predicted no infection.

As noted in a previous paragraph, there were 30 cases (of 128) in which the model did not predict a real infection. These missed infections occurred in 9 of 11 locations (Table 3) and accounted for only 8.1% of the total rescaled disease found in bait plants; the average rescaled disease value was 0.37 (with a 95% confidence interval of 0.30 to 0.43) for P + O + and was 0.10 (0.08 to 0.13) for P−O+, with only 1 (specifically at SPIL05) of 30 cases showing a high rescaled disease value (shown as an outlier in Figure 2). At SPIL05 (Table 3), there was an average of 1.1 lesions per plant in bait plants exposed for 48 h between May 19 and 20; that corresponds to a relative disease value of 0.39, and 2.8 was the highest number of lesions per plant found on plants exposed for 72 h between May 14 and 16 (Figure 3). During the May 19 and 20 exposure period, there was no rain and only 4 h of wetness, and average T and RH were 11.1 °C and 72.6%, respectively, at the Pullman Airport, which was 6 km from the SPIL experimental site. Because the model assumes that airborne ascospores of *A. rabiei* are scrubbed from the air and deposited on the plant surface by a minimum of 1 mm of rain, the model did not calculate infection in all cases in which there was no rain or <1 mm of rain.

#### 2.4.2. Validation of Disease Progress

**Figure 4:**
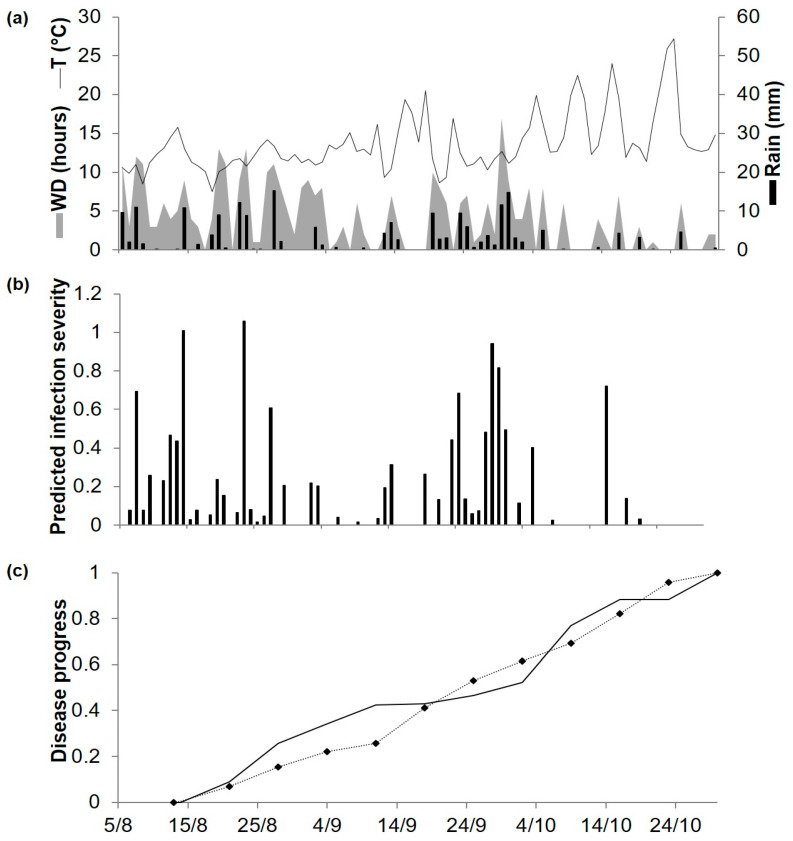
Predicted and observed disease progress (Ascochyta blight) on cv. Kaniva at the experimental field at the University of Adelaide’s Waite Campus, Australia, in 1996. (**a**) Weather variables: air temperature (T, °C, solid line), rainfall (Rain, mm, black bars), and leaf wetness duration (WD, hours, grey area). (**b**) Bars represent the infection severity predicted by the model. (**c**) Solid line represents the accumulated infection severity predicted by the model and rescaled to the final value of the year; the dotted line represents the observed Ascochyta blight severity rescaled to the final value of the year (71%).

As noted earlier, predicted and observed disease progress were compared at ADE96, ADE97, ADE98, and POG19. At ADE96, the daily temperature ranged from 7 to 24 °C, and rainfall was regularly distributed but was reduced in the last month (Figure 4a). The model predicted repeated infections until early October, which accounted for 77% of the seasonal disease severity predicted by the model; only 3 further infections were predicted until late October (Figure 4b). The disease was first observed on 14 August and then increased almost linearly over time, with a final disease severity of 71% of affected leaf and stem area (Figure 4c). For the goodness-of-fit of predicted versus observed data, concordance correlation coefficient (CCC) = 0.957 and root mean square error (RMSE) = 0.087. The model showed a tendency toward overestimation (coefficient of residual mass (CRM) = −0.059).

**Figure 5:**
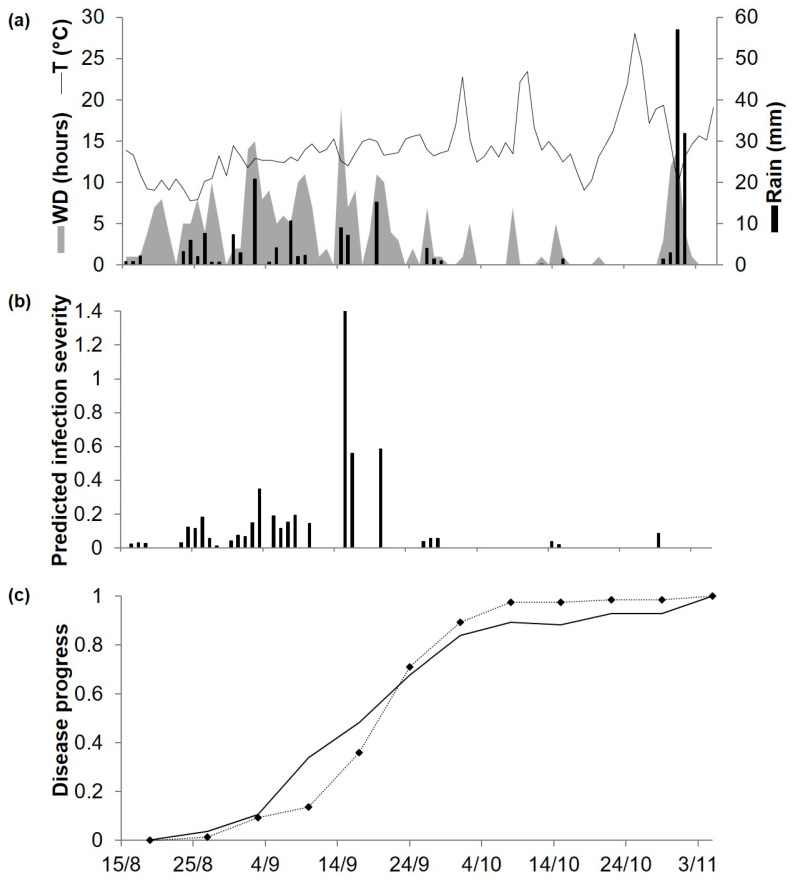
Predicted and observed disease progress (Ascochyta blight) on cv. Kaniva at the University of Adelaide’s Waite Campus, Australia, in 1997. (**a**) Weather variables: air temperature (T, °C, solid line), rainfall (Rain, mm, black bars), leaf wetness duration and (WD, hours, grey area). (**b**) Bars represent the infection severity predicted by the model. (**c**) Solid line represents the accumulated infection severity predicted by the model and rescaled to the final value of the year. Dotted line represents the accumulated value of Ascochyta blight severity rescaled final value of the year (80%).

At ADE97, temperatures were quite similar to those at ADE96, but the rain distribution was different; frequent rain events in the first 7 weeks were followed by a dry period that lasted until the last week, when heavy rains were recorded (Figure 5a). The model predicted repeated infection periods between 16 August and 10 September, which caused a rapid increase of the predicted disease progress curve; at the beginning of October, accumulated severity of predicted infections accounted for about 90% of the predicted seasonal disease severity (Figure 5b). Disease symptoms were first observed on 20 August, and disease severity increased progressively until the last day of assessment, reaching 80% of affected plants area (Figure 5c). For the goodness-of-fit of predicted versus observed data, CCC = 0.972 and RMSE = 0.087. The model showed a slight tendency toward overestimation (CRM = −0.001).

**Figure 6:**
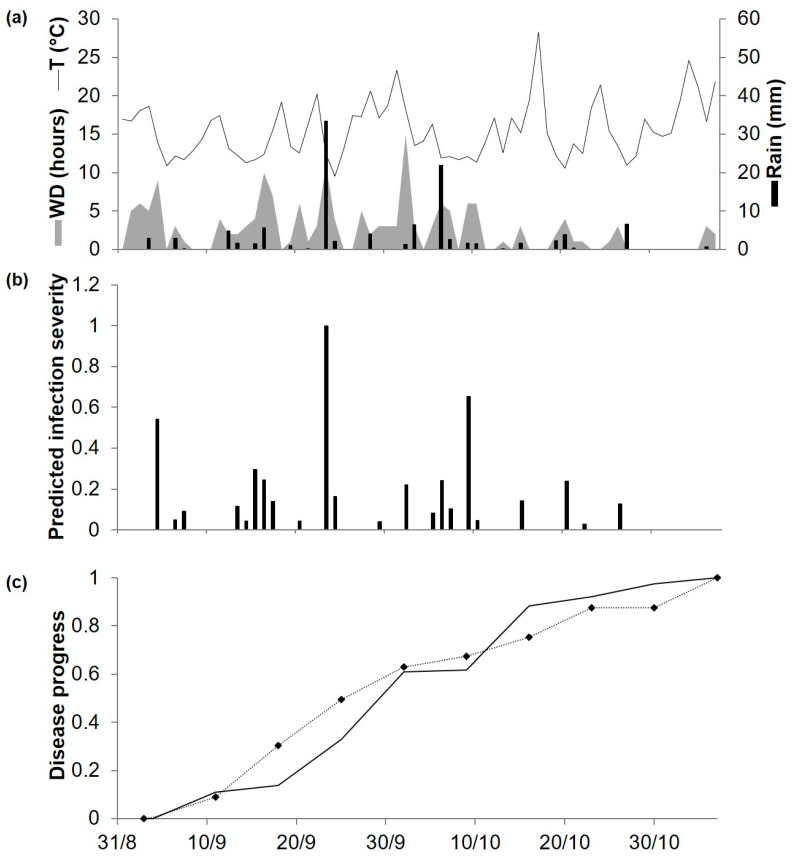
Predicted and observed disease progress (Ascochyta blight) on cv. Kaniva at the University of Adelaide’s Waite Campus, Australia, in 1998. (**a**) Weather variables: air temperature (T, °C, solid line), rainfall (Rain, mm, black bars), leaf wetness duration and (WD, hours, grey area). (**b**) Bars represent the infection severity predicted by the model. (**c**) Solid line represents the accumulated infection severity predicted by the model and rescaled to the final value of the year. Dotted line represents the accumulated value of Ascochyta blight severity rescaled final value of the year (40%).

At ADE98, temperatures were similar to those recorded in previous years. During the trial, the weekly amount of rain was < 1 mm, except that 5 mm of rain fell during one week in September (Figure 6a). The model predicted 22 infection periods distributed throughout the period, predicting a regular progress of the disease (Figure 6b,c). Disease assessment began on 21 August, but Ascochyta blight symptoms were not observed until September 4; afterwards, the disease increased gradually (Figure 6c) to a final observed disease severity of about 40% of affected plant area, which was lower than in the two previous years. For the goodness-of-fit of predicted versus observed data, CCC = 0.949 and RMSE = 0.098. The model showed a slight tendency toward underestimation (CRM = 0.020).

**Figure 7:**
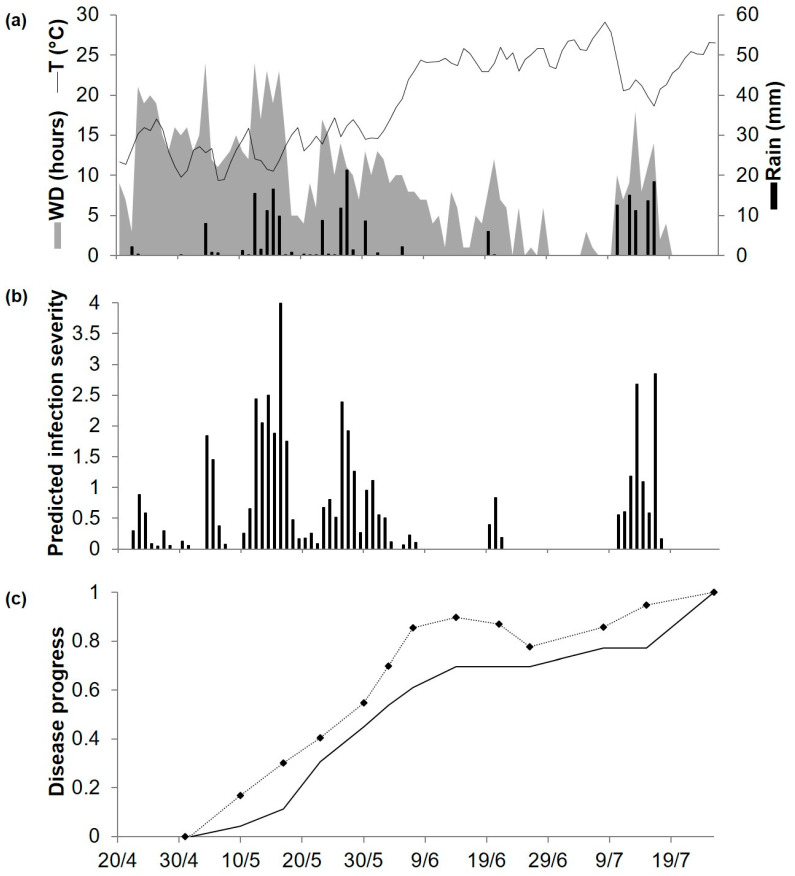
Predicted and observed disease progress (Ascochyta blight) on cv. Sierra at Poggiorsini, Southern Italy, in 2019. (**a**) Weather variables: air temperature (T, °C, solid line), rainfall (Rain, mm, black bars), leaf wetness duration and (WD, hours, grey area). (**b**) Bars represent the infection severity predicted by the model. (**c**) Solid line represents the accumulated infection severity predicted by the model and rescaled to the final value of the year. Dotted line represents the observed accumulated value of Ascochyta blight severity rescaled final value of the year (93%).

At POG19, which has a Mediterranean climate, spring was mild (average daily temperature of 16–18 °C) and summer was hot (average daily temperature of 24–28 °C), with a total of 202.4 mm of rain occurring mainly in May and mid-July (Figure 7a). Model calculations began on 20 April, and the model predicted infection on 59 days, 42 of which were between May and early June and accounted for about 60% of the total predicted disease severity (Figure 7b). Ascochyta blight lesions on chickpea plants were first observed on 2 May (Figure 7c). The disease increased rapidly until about 10 June and at lower rates afterwards; final observed disease severity was 93% of affected leaf and stem area. For the goodness-of-fit of predicted versus observed data, CCC = 0.893 and RMSE = 0.129. The model showed a slight tendency toward underestimation (CRM = 0.159).

**Figure 8:**
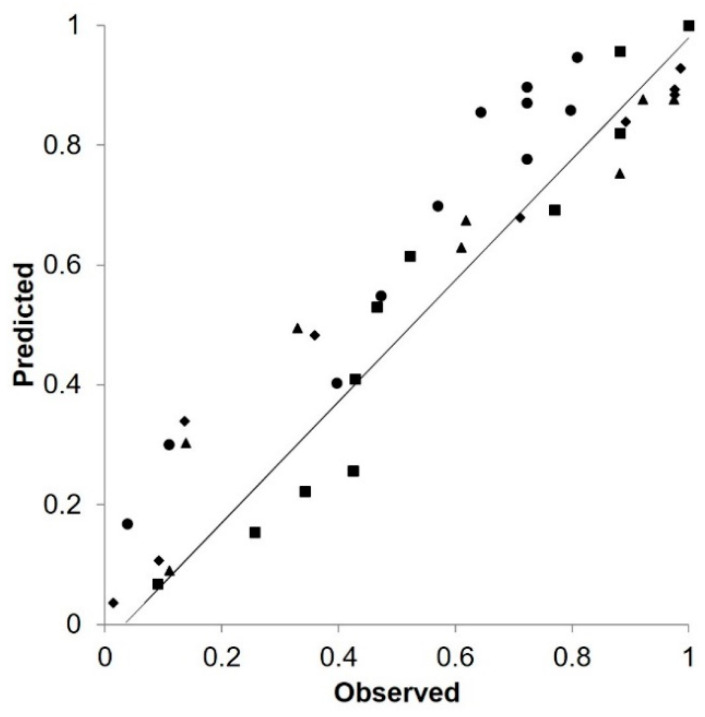
Plots of predicted versus observed values of Ascochyta blight severity for four monitoring locations and years: Poggiorsini, Southern Italy, in 2019 (●); University of Adelaide’s Waite Campus, Australia, in 1996 (■); University of Adelaide’s Waite Campus, Australia, in 1997 (♦); and University of Adelaide’s Waite Campus, Australia, in 1998 (▲). Predicted refers to the predicted disease infection severity through the growing season and rescaled to the final value of the year. Observed refers to the observed disease severity rescaled to the final value of the year.

For disease progress, an overall comparison of predicted versus observed values at ADE96, ADE97, ADE98, and POG19 (Figure 8) gave CCC = 0.947, with little average distance between the observed data and the fitted line, i.e., RMSE = 0.103. This indicated that the model can be considered reliable. The model showed a slight tendency toward underestimation (CRM = 0.041).

## 3. Discussion

In this study, we developed a mechanistic model for Ascochyta blight of chickpea by using the available knowledge on the pathogen and disease. According to Rossi et al. [41], application of systems analysis to the literature is useful for conceptualizing pathosystems and developing plant disease models; the case study of black-rot of grapes showed how published information and data can be used to develop mechanistic, dynamic, weather-driven models [41]. Unlike previous models for Ascochyta blight [9,31,33,34,35,36], the model developed in the current research considers the entire life cycle of *A. rabiei* and accounts for the susceptibility of the host plant at different growth stages. Changes in plant susceptibility during the season have substantial effects on polycyclic diseases, and the susceptibility of chickpea to *A. rabiei* is known to increase with host stage and to be highest at pod formation stage [21,22,23,49].

Using a systematic literature search, we obtained detailed information on the *A. rabiei* life cycle and data on the effect of weather conditions (i.e., air temperature, rain, and leaf wetness duration) on infection from 82 papers. Our literature search also revealed some incomplete knowledge about important biological and epidemiological aspects of the pathogen and the disease. In developing a model, we dealt with these knowledge gaps by making explicit assumptions and/or by using data for plant pathogens that belong to the same taxonomic group as *A. rabiei*. The model was validated against independent data, and this enabled an indirect evaluation of the validity of model assumptions: the accurate predictions of Ascochyta blight epidemics suggest that either the assumptions were correct or if incorrect, did not greatly reduce the ability of the model to make correct predictions.

For the primary inoculum, the model makes four assumptions: (i) ascospores from pseudothecia that had overwintered in infested chickpea debris in neighboring fields are the sole relevant source of primary inoculum; (ii) the ascospores are airborne during the primary inoculum season; (iii) the ascospores are scrubbed from the air by rain and are deposited on the crop surface; and (iv) this ascospore dose cannot be quantified and is therefore assumed to be constant and equal to 1, so that the further development of the infection is expressed as a proportion of this ascospore dose. These assumptions could result in false positive predictions of infection if no ascospores are deposited on the crop when predicted by the model, or in false negative predictions if ascospores are deposited on the crop when not predicted. Of the 232 total cases used to validate the primary infection compartment of the model, there were 19 cases (5.2%) in which the model predicted infections that were not observed (FPP = 0.18) and 30 cases (11.9%) in which the model failed to predict infections that were observed (FNP = 0.23).

The first kind of error (FPP) does not affect crop health but can lead to needless fungicide applications. Reducing this error requires a better estimate of the presence of ascospores. If spore traps were used to detect the presence of *A. rabiei* ascospores, a model run would begin only when inoculum was present and ASCMAT had been assessed. The use of spore traps for the monitoring of airborne inoculum for supporting epidemiological models has been suggested for other pathosystems [63,64]. As an alternative to the use of spore traps, an additional model compartment could be developed that predicts the dynamics of pseudothecia formation and ascospore maturation, as has been done for *V. inaequalis* [65], *Gibberella zeae* [66], *L. maculans* [67], and *Guignardia citricarpa* [68]. Unfortunately, the data currently available in the literature are inadequate for developing such a model compartment for *A. rabiei*, i.e., studies are needed on *A. rabiei* pseudothecia formation and ascospore maturation.

The second type of error (FNP) leads to real infections being missed and reduces the model’s usefulness, because growers would fail to protect crops when necessary. To reduce this error, researchers should assess the deposition of ascospores on the crop during weak rain events or in the absence of rain. To avoid this error, we also considered reducing the rainfall threshold used by the model to predict ascospore deposition (by using ≥0.2 or 0.6 mm h^−1^), but this reduction led to an increase of FPP that significantly decreased the overall accuracy of the model (not shown). In addition, the rain data used for model validation were measured in airport weather stations that were located up to 17 km from experimental sites; it is possible that rain fell on the bait plants but not at the airports. The problem does not seem to be serious, however, in that the disease corresponding to FNP accounted for only 8.1% of the total disease found on bait plants, i.e., when the model failed to predict an infection that occurred, the infection only resulted in light disease.

In addition to making assumptions about the primary inoculum, we also made the following three assumptions about the secondary inoculum: i) Ascochyta blight lesions continue to produce conidia for the entire epidemic; ii) these conidia are dispersed by rain splashes whenever there is rain; and iii) this dose of conidia cannot be quantified and is, therefore, kept constant and = 1, so that the further development of the disease is expressed as a proportion of this dose of conidia. These assumptions could result in an overestimation of the presence of secondary inoculum and, ultimately, an overestimation of the epidemic. The model, however, slightly underestimated real disease severity, i.e., the overall CRM value for the four validation data sets was + 0.041.

Despite the errors, the model provided a reliable representation of Ascochyta blight epidemics, with overall accuracy = 0.79 for primary infections and CCC = 0.947 for disease progress. Considering that the model was validated by using independent data that were collected in multiple years in sites with different climates (i.e., Pacific Northwest climate at Washington, and Mediterranean climate in Israel, Spain, Australia, and South Italy), and that involved a wide range of disease severity, the model may be considered to be accurate (i.e., it provided predictions close to reality) and robust (i.e., it provided accurate predictions in a range of environments and epidemiological conditions) [40]. Nevertheless, gaps that affected model accuracy were identified in our current knowledge of the biology and epidemiology of *A. rabiei*. For example, the model could be improved by further studies of pseudothecia formation and ascospore maturation. We are currently evaluating the use of the model for scheduling fungicide applications and for supporting farmers in their decision-making about disease control.

## 4. Materials and Methods

### 4.1. Literature Search

A literature search was conducted to collect original data on the biology, ecology, and epidemiology of *A. rabiei* as well as the data on the interaction between the pathogen and chickpea. The search was carried out in the World Wide Web and in the following databases: CAB Abstract (http://www.cabdirect.org accessed on 14 May 2019), Google Scholar (https://scholar.google.it accessed on 22 May 2019), Scopus (https://www.scopus.com accessed on 16 May 2019), and Web of Science (https://apps.webofknowledge.com accessed on 17 May 2019). The following combinations of keywords were used: (i) *Ascochyta rabiei* OR *Didymella rabiei* OR synonyms; (ii) Ascochyta blight OR other common names; and (iii) life cycle OR inoculation OR germination OR penetration OR appressoria OR infection OR survival OR incubation OR latent period OR pycnidia OR conidia OR pycnidiospores OR pseudothecia OR ascospores OR overwintering OR model OR prediction. Papers were first examined and selected on the basis of the information in the title and abstract. The full text of each selected paper was then read and reviewed. Additional papers were selected from the References of the selected papers; these papers were also retrieved and reviewed.

### 4.2. Systems Analysis and Model Development

The information retrieved in the selected papers was used to conceptualize the model, as indicated by Rossi et al. [40], and a relational diagram was drawn, representing the system structure and dynamics. The relational diagram included state variables, flows, rate variables, driving variables, and switches. The life cycle of *A. rabiei* was divided into state variables, i.e., variables that represent the state of the system (e.g., mature ascospores generated in overwintering pseudothecia on chickpea debris, dose of viable ascospores and conidia landing on plant surfaces, infection sites, and lesions). The flows from one state to the following one were governed by rate variables depending on external, driving variables (e.g., environmental conditions and chickpea growth stage) or switches accounting for logical operators with the syntax if ‘condition’ then ‘go to’, else ‘go to’.

The dynamics of the system were regulated by mathematical equations relating external, influencing variables (i.e., weather data and chickpea growth stage) to rates. Mathematical equations linking the weather variables to rate variables were developed from the literature. Data on the pathogen or the disease were obtained directly from the text, tables, or graphs in the papers; the GetData Graph Digitizer 2.24 (http://getdata-graph-digitizer.com accessed on 3 July 2019) was used to obtain precise data from graphs. Data were then fit with proper mathematical equations, which were selected based on the shape of the data and the Akaike information criterion [69]; equations that provided the smallest AIC values were considered the most likely to be correct. Equation parameters were estimated using the non-linear regression procedure of SPSS (IBM SPSS Statistics 25, IBM Corp, Armonk, NY, USA), which uses the Levenberg–Marquardt algorithm to minimize the residual sums of squares. The goodness-of-fit was then evaluated by means of the standard errors of parameters, the distribution of residuals of predicted versus observed values, and adjusted R^2^. Further information on the development of mathematical equations is provided in the Supplementary material.

### 4.3. Model Validation

#### 4.3.1. Primary Infections

Data used for validation of Ascochyta blight primary infections were retrieved from Chilvers et al. [70], Gamliel-Atinsky et al. [71], and Trapero-Casas et al. [72].

Chilvers et al. [70] exposed bait plants of the susceptible chickpea cv. Burpee to the pathogen at two sites, Washington State University’s Spillman Research Farm, US (46°41′ N, 177°08′ W) and the Washington State University campus at Pullman, US (46°43′ N, 117°09′ W), both of which were under a Pacific Northwest climate. At each site for two consecutive years (2004 and 2005), bait plants grown in isolation in a greenhouse were placed within 1 m of overwintered chickpea debris affected by *A. rabiei* for 2 or 3 days, and were then returned to the greenhouse and replaced with new plants; Ascochyta blight lesions were counted on each plant 2 weeks after exposure. Experiments were conducted between 5 April and 26 May in 2004, and between 28 February and 25 May in 2005. Bait plants that were exposed after these periods were not considered because, according to the authors, the depletion of inoculum reservoir and irrigation probably affected the infection occurrence. Weather data were obtained from the Pullman Airport weather station (KPUW, 46°44′ N, 117°06′ W), which was located about 6.0 km from the Spillman Research Farm and about 3.5 km from the University campus. The combination of locations and years are hereafter referred to as acronyms with three letters for the location and two numbers for the year, i.e., SPIL04 and SPIL05 for the Spillman Research Farm and PUL04 and PUL05 for the campus at Pullman.

Gamliel-Atinsky et al. [71] exposed bait plants of the susceptible chickpea cv. Sfaradit to the pathogen at the Central Experimental Station of the Agricultural Research Organization, Bet Dagan, Israel (31°59′ N, 34°49′ E), under a Mediterranean climate. Bait plans grown in isolation were placed outdoors close to *A. rabiei*-affected overwintered chickpea debris from 20 January to 30 April in 1996, and from 22 February to 29 April in 1998 (acronyms BET96 and BET98). After being exposed for 3 or 4 days, bait plants were moved to a greenhouse, and Ascochyta blight lesions were assessed after 14 to 18 days. Weather data were obtained from the Ben Gurion Airport (LLBG, 32°00′ N, 34°53′ E), which was located about 17 km from the experimental site.

Trapero-Casas et al. [72] exposed chickpea bait plants cv. Blanco Lechoso to the pathogen at the Alameda del Obispo research farm, Cordoba, Spain (37°51′ N, 4°47′ W), under a Mediterranean climate. Bait plants were placed near *A. rabiei*-affected overwintered chickpea debris at weekly intervals from early January to the end of March for a 5-year period (1988 to 1992) (acronyms COR88 to COR92). After exposure, plants were transferred to a greenhouse and were assessed for disease 2 weeks later. Bait plants exposed after the end of March were not considered because, according to the authors, the exhaustion of primary inoculum in debris meant that reliable data could not be obtained. Weather data were retrieved from the Cordoba airport (LEBA, 37°50′ N, 4°50′ W), which was located about 3 km from the experimental site.

Because the disease data collected in these three papers were expressed in different units of measure (i.e., average number of lesions per plant per day, disease severity, or disease incidence), disease records of each experiment were rescaled to their maximum to obtain relative disease data expressed on a scale from 0 to 1. This transformation made the comparison of the disease among years and locations possible.

For validation purposes, the model was run using weather data in each year and location starting from the first day in which bait plants were exposed. The correspondence between model predictions and real Ascochyta blight infections during the periods of plant exposure was evaluated through a Bayesian analysis [73,74]. Periods in which infections were predicted by the model were considered as positive outcomes (i.e., P+; an infection is predicted); similarly, periods in which no infection was predicted were considered as negative outcomes (i.e., P−). Real infections were considered to occur when disease symptoms were observed on bait plants (i.e., O +; an infection was observed), or were assumed not to occur when disease symptoms did not appear on bait plants (i.e., O−). Therefore, any period in which bait plants have been exposed (case) was classified as follows: (i) true positive, i.e., P + O +; (ii) true negative, i.e., P−O−; (iii) false positive, i.e., P + O−; or (iv) false negative, i.e., P−O + . A 2 × 2 contingency table was then organized showing the true positive proportion (TPP or sensitivity), the true negative proportion (TNP or specificity), the false positive proportion (FPP), and the false negative proportion (FNP).

Prior probabilities for the disease to occur, P(O + ), or not to occur, P(O−), were calculated and compared with the following posterior probabilities: (i) there was infection when predicted by the model, *P*(P + O + ); (ii) there was no infection when not predicted, *P*(P−O−); (iii) there was no infection when predicted (i.e., unjustified alarms), *P*(P + O−); and (iv) there was infection when not predicted (i.e., missed real infections), *P*(P−O + ). Positive and negative likelihood ratios (LR+ and LR−, respectively) were calculated by dividing TPP by FPP and FNP by TNP, respectively. The Youden index (J) was used to evaluate the model performance in avoiding wrong predictions. Finally, the ratio between right and total predictions was calculated as an indicator of overall model accuracy.

#### 4.3.2. Disease Progress

To validate the ability of the model to predict epidemic development through the season caused by the concatenation of infection cycles, we used disease progress curves reported by Khan [59] and disease assessments carried out by Meriggi et al. (see Acknowledgements) in a field located at Poggiorsini, Southern Italy, in 2019.

Khan [59] conducted field assessments in 3 years, 1996 to 1998, at the University of Adelaide’s Waite Campus (34°58′ S, 138°38′ E) (acronyms ADE96 to ADE98). Disease severity (%) was assessed weekly on cv. Kaniva (moderately susceptible) from 14 August to 30 October 1996, 20 August to 5 November 1997, and 4 September to 6 November 1998. In each year, disease on 30 plants was assessed as described by Gowen et al. [75], who rated disease severity on a scale from 0 to 100% in 10% steps, with 0 to 10% indicating “no infection–small lesions” and 100% indicating “completely dead plants”. Weather data were obtained from the Adelaide Airport (YPAD, 34°56′ S, 138°32′ E), which was located about 13 km from the Waite Campus.

In a chickpea field at Poggiorsini (40°54′ N 16°15′ E) (acronym POG19), Meriggi et al. assessed disease severity (%) on the susceptible cv. Sierra weekly from 10 May to 26 July 2019. Ten groups of 4 plants were designated to assess the change in disease level on the same individuals during the growing season. The percentage of disease severity was assessed using the following scale: 0: no lesions; 1: few lesions, affected area <5%; 2: several lesions, affected area 5−20%; 3: affected area 21–40%; 4: affected area 41–60%; 5: affected area 61–80%; and 6: affected area >80%. The weather data were recorded by a station (PESSL iMetos 3.3) located 3.5 km from the experimental field.

The model was operated starting from the day of the last assessment in which no disease was observed. Both predicted infection severity and observed disease severity were accumulated during each disease assessment period and were rescaled to their final value; values were rescaled from 0 to 1 to make the data collected in different experiments comparable [76,77]. For the evaluation of model performance, the root mean square error (RMSE), the coefficient of residual mass (CRM), and the concordance correlation coefficient (CCC) were calculated [78,79]. RMSE is the measure of the average distance occurring between the real data and the fitted line [79]. CRM represents the tendency of the model toward over or underestimation; a negative CRM indicates that the model overestimates, and a positive CRM indicates that the model underestimates [79]. CCC estimates the difference between the fitted line and the perfect agreement line; a CCC value of 1 indicates perfect agreement [78].

## Figures and Tables

**Table 1:** List of variables, rates, and parameters used in the model, and their units.

Acronym	Description	Unit
ASCDOSE	Dose of viable ascospores landing on plant surface	0/1
*ASCINF*	Rate of ascospore infection	0 to 1
*ASCINF*(T)	Rate of ascospore infection depending only on T	0 to 1
*ASCINF*(WD)	Cumulative proportion of ascospores infection depending only on WD	0 to 1
*ASCINF*’(WD)	Rate of ascospore infection depending only on WD	0 to 1
ASCMAT	Mature ascospores generated in overwintering pseudothecia on chickpea debris	0/1
CONDOSE	Dose of viable conidia landing on plant surface	0/1
*CONINF*	Rate of conidia infection	0 to 1
*CONINF*(T)	Rate of conidia infection depending only on T	0 to 1
*CONINF*(WD)	Cumulative proportion of conidia infections depending only on WD	0 to 1
*CONINF’*(WD)	Rate of conidia infection depending only on WD	0 to 1
CONMAT	Mature conidia produced by pycnidia in lesions	0/1
f(T)	Equation accounting for the influence of temperature in each i^th^ hour during INC or LAT	0 to 1
GS	Correction factor accounting for chickpea growth stage	N
Hdry	Consecutive hours of dryness	N hours
INC	Incubation period, i.e., the period from infection to VLES onset	0 to 1
INCmin	Minimum number of hours required for symptoms appearance at any temperature	N
LAT	Latency period, i.e., the period from infection to CONMAT onset	0 to 1
LATmin	Minimum number of hours required for pycnidia production at any temperature	N
R	Hourly rainfall	mm
SEV1	Severity of the primary infection	0 to 1
SEV2	Severity of the secondary infection	0 to 1
*SUR*	Survival rate of ascospores and conidia	0 to 1
T	Hourly air temperature	°C
Teq	Equivalent of temperature calculated as (T – Tmin)/(Tmax – Tmin)	0 to 1
Tmax	Maximum temperature for infection of ascospores or conidia, or incubation or latency progress	°C
Tmin	Minimum temperature for infection of ascospores or conidia, or incubation or latency progress	°C
Topt	Optimum temperature for infection of ascospores or conidia, or incubation or latency progress	°C
VLES	Visible lesions produced by ascospores or conidia infections	0/1
WD	Wetness duration, i.e., duration of the wet period	N hours

**Table 2:** GS values for three chickpea growth stages.

Growth Stage	GS
vegetative	0.857
flowering	0.942
pod formation	1.000

**Table 3:** Experimental sites (acronyms for locations and years) used to validate model predictions of primary infection. Periods of bait plant exposure were classified as follows: TPP = true positive proportion (sensitivity), TNP = true negative proportion (specificity), FNP = false negative proportion, and FPP = false positive proportion.

Experimental Sites	Total Number of Cases 232					
	TPP	TNP	FNP	FPP	P + O+ Disease Range ^d^	P – O + Disease Range ^e^
COR88	8	1	0	3	2–43 ^a^	
COR89	7	4	1	0	0.3–41 ^a^	0.3 ^a^
COR90	5	3	3	1	19–39 ^a^	0.2–5 ^a^
COR91	8	2	0	2	0.3–34 ^a^	
COR92	3	6	1	2	11–43 ^a^	0.3 ^a^
BET96	14	7	5	4	2–84 ^b^	4–31 ^b^
BET98	5	11	3	1	4–98 ^b^	4–9 ^b^
SPIL04	12	9	2	0	0.2–6.3 ^c^	0.04–2.3 ^c^
PUL04	9	10	1	3	0.02–1.8 ^c^	0.04 ^c^
SPIL05	14	13	9	2	0.1–2.8 ^c^	0.01–1.1 ^c^
PUL05	13	17	5	3	0.04–1.4 ^c^	0.02–0.4 ^c^

^a^ Disease severity (%). ^b^ Disease incidence (%). ^c^ Lesion per plant per day. ^d^ Range of disease assessed when infection was predicted by the model. ^e^ Range of disease assessed when infection was not predicted by the model.

**Table 4:** Comparison between *Ascochyta rabiei* primary infections predicted by the model and observed on bait plants, and corresponding properties of the model.

		Predicted Infection			Prior Probability (P)	Posterior Probability (P)		
		Yes (P + )	No (P−)	Total				
**Observed infection**	Yes (O + )	98 TPP ^a^ = 0.77	30 FNP ^b^ = 0.23	128	P(O + ) = 0.55	*P*(O + P + ) = 0.838		*P*(O + P−) = 0.162
	No (O −)	19 FPP ^c^ = 0.18	85 TNP ^d^ = 0.82	104	P(O − ) = 0.45	*P*(O−P + ) = 0.224		*P*(O−P−) = 0. 776
	Total	117	115	232				
**Likelihood ratio (LR)**	LR( + ) = TPP / FPP = 4.19							
	LR(−) = FNP / TNP = 0.29							
**Youden index**	J = TPP – FPP = 0.58							
**Overall accuracy** ^e^	0.79							

^a^ True positive proportion (sensitivity). ^b^ False negative proportion. ^c^ False positive proportion. ^d^ True negative proportion (specificity). ^e^ Calculated by dividing the number of correct predictions by the total number of predictions.

## Data Availability

Please refer to suggested Data Availability Statements in section “MDPI Research Data Policies” at https://www.mdpi.com/ethics.

## Supplementary Materials

The following are available online at https://www.mdpi.com/2223-7747/10/3/464/s1, Figure S1. Relationship between relative infection severity of Ascochyta rabiei and temperature (°C). Dots show the data of Trapero-Casas and Kaiser [46], and the dotted line shows the fit of data using the bete equation (1) [47], with R^2^ = 0.971. Figure S2. Relationship between relative infection severity of Ascochyta rabiei and wetness duration (hours). Dots show the data of Trapero-Casas and Kaiser [46], and the dotted line shows the fit of data with equation (2), with R^2^ = 0.995. Figure S3. Dynamic of Ascochyta rabiei survival of ascospores and conidia. Dots show the data of Trapero-Casas and Kaiser [46], and the dotted line shows the fit of data with equation (3), with R^2^ = 0.937. Figure S4. Effect of temperature on length of (a) incubation and (b) latency periods, expressed in hours after infection by Ascochyta rabiei. In (a) symbols show data of Basandrai et al. [22] (▲), Trapero-Casas and Kaiser [49] (●), and Chauhan and Sinha [50] (■); the dotted line shows the length of incubation as predicted by the equation of Magarey et al. [48] (6), with R^2^ = 0.887. In (b) dots show the average number of hours required for pycnidia formation as calculated using data of Trapero-Casas and Kaiser [49]; the dotted line shows the length of the incubation as predicted by the equation of Magarey et al. [48] (7), with R^2^ = 0.945. Figure S5. Relationship between relative infection severity of Ascochyta rabiei and temperature (°C). Symbols show the data of Trapero-Casas and Kaiser [46,49] (●), Khan [59] (▲), and Weltzien and Kaack [60] (■); the dotted line shows the fit of data using a bete equation (9) [47], with R^2^ = 0.978. Figure S6. Relationship between relative infection severity of Ascochyta rabiei and wetness duration (hours). Symbols show the data of Trapero-Casas and Kaiser [46,49] (●), Armstrong-Cho et al. [61] (■), Khan [59] (▲), and Jhorar et al. [62] (♦); the dotted line shows the fit of data with equation (10), with R^2^ = 0.944.
