# Supplementary material for: A Mechanistic Weather-Driven Model for Ascochyta rabiei Infection and Disease Development in Chickpea
Source: Plants (Basel). 2021 Mar 1;10(3):464. doi: 10.3390/plants10030464 (PMC8000037; doi:10.3390/plants10030464)
Supplement: Supplementary file 1 [file plants-10-00464-s001.zip › Supplementary/Salotti and Rossi Supplementary.docx]

Development of model equations

The supplementary material describes how data from the literature were used to develop the model equations.

Equation (1)

Equation (1) of the model was developed using data of Trapero-Casas and Kaiser [1]. Two-week-old chickpea seedlings of cv. Burpee (susceptible) that had five to seven leaves were inoculated with an ascospore suspension (2 x 10^4^ ascospores per ml) using an airbrush sprayer. Plants were sprayed until runoff and were incubated in plastic moist chambers that were placed in darkened environmental chambers set at 5, 10, 15, 20, 25, or 30°C. After 48 h, plants were removed from the plastic cages and returned to environmental chambers with a 14-h light period or were moved to a greenhouse at 18-26°C for symptom development. Disease severity was assessed using a 0-9 rating scale that described the percentage of disease from 0 (no infection) to 100% (dead plant). Average disease severity at each temperature was calculated using values recorded on plants that were incubated both in environmental chambers and in a greenhouse. The average disease severity was then rescaled relative to the maximum value to obtain data expressed on a 0-to-1 scale, giving the relative infection severity. These data (dependent variable Y) were then regressed against temperature by using a bete equation [2] in the form: Y = (a X^b^ (1-X))^c^, where the independent variable X is an equivalent of temperature (Teq) calculated as Teq = (T-Tmin) / (Tmax-Tmin). Tmin and Tmax were defined based on a spore germination experiment reported in Trapero-Casas and Kaiser [1]. In this experiment. ascospores were discharged from fruiting bodies directly onto water agar and were kept at 100% relative humidity in the dark at temperatures between 0 and 35°C. The percentage of spores that germinated was determined by observing 60 random ascospores; because no ascospores germinated at 0°C or 35°C, Tmin was set at 0°C and Tmax was set at 35°C. The best fit of the experimental data was obtained with a = 4.929±0.200, b = 1.360±0.068, and c = 4.663±0.830, with R^2^=0.971 (Figure S1).


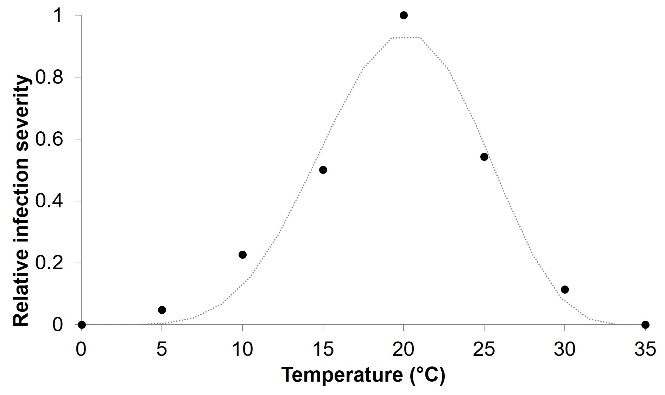


**Figure S1.** Relationship between relative infection severity of *Ascochyta rabiei* and temperature (°C). Dots show the data of Trapero-Casas and Kaiser [1], and the dotted line shows the fit of data using the bete equation (1) [2], with R^2^ = 0.971.

Equation (2)

Equation (2) of the model was developed from an analysis of data retrieved from Trapero-Casas and Kaiser [1]. Two-week-old chickpea seedlings of cv. Burpee (susceptible) were sprayed until runoff with an ascospore suspension (2 x 10^4^ ascospores per ml) and were incubated in a moist chamber at 20°C. After 0, 6, 12, 24, or 48 h, plants were removed from the moist chamber, dried with a cool air flow, and placed in a growth chamber at 20°C with a 14-h light period and a relative humidity (RH) <50% for 7 days. Afterwards, plants were transferred to a greenhouse and kept at 18-26°C. Disease was assessed 14 days after inoculation by using a 0-9 rating scale that described the percentage of disease severity from 0 (no infection) to 100% (dead plant). These data were rescaled relative to the maximum value to obtain data that were expressed on a 0-to-1 scale (dependent variable Y) and that were then regressed against wetness duration (independent variable X). The best fit of the experimental data was obtained using a linear equation Y = a X + b, with a = 0.021±0.001, b = 0.009±0.020, and R^2^= 0.995. Relative infection severity as a function of wetness duration is shown in Figure S2. The first-order derivative of this equation was used in equation (4) of the model.

**
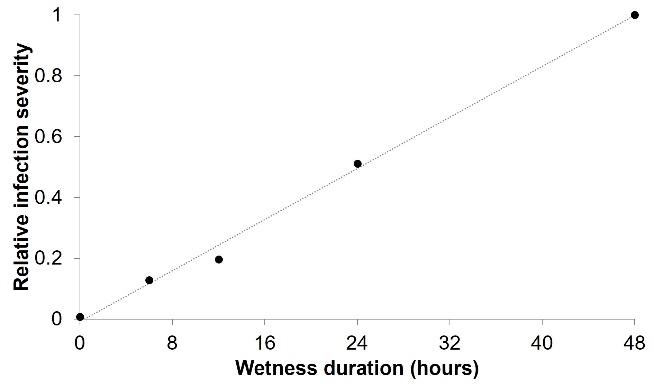
**

**Figure S2.** Relationship between relative infection severity of *Ascochyta rabiei* and wetness duration (hours). Dots show the data of Trapero-Casas and Kaiser [1], and the dotted line shows the fit of data with equation (2), with R^2^ = 0.995.

Equation (3)

The model calculates the survival rate of both ascospores and conidia with equation (3), which was developed from data of Trapero-Casas and Kaiser [1], who obtained the data with the following experiment. Two-week-old chickpea seedlings of cv. Burpee (susceptible) were sprayed until runoff with a spore suspension (2 x 10^4^ ascospores or conidia per ml). Plants were kept at 20°C and subjected to a 24-h interrupted-wet period with 6-h wetness immediately after inoculation and 18-h wetness after a dry period of 6, 12, 24, or 48 h. During the dry period, plants were dried under a cool air flow and then kept in a growth chamber at RH <50%. These plants were then rewetted by spraying their leaves and stems with water. After the second wet period, plants were dried and incubated for 7 days in growth chambers and then transferred to a greenhouse until the onset of symptoms. Fourteen days post inoculation, disease severities were recorded using a 0 (no symptoms, 0% affected foliage)-to-9 (dead plant, 100% affected foliage) rating scale. Because the ability to survive an intermittent dry period during the infection process was similar for both ascospores and conidia in the experiment of Trapero-Casas and Kaiser [1], we developed a single equation that accounted for survival of both spore types. Data retrieved from the paper were first rescaled to the maximum, giving the relative survival of spores (dependent variable Y), which was then regressed against the number of hours of the dry period (independent variable X) by using a linear equation in the form Y = 1 - a X. The best fit of the data (Figure S3) was achieved with a = 0.017±0.001, with R^2^=0.937.


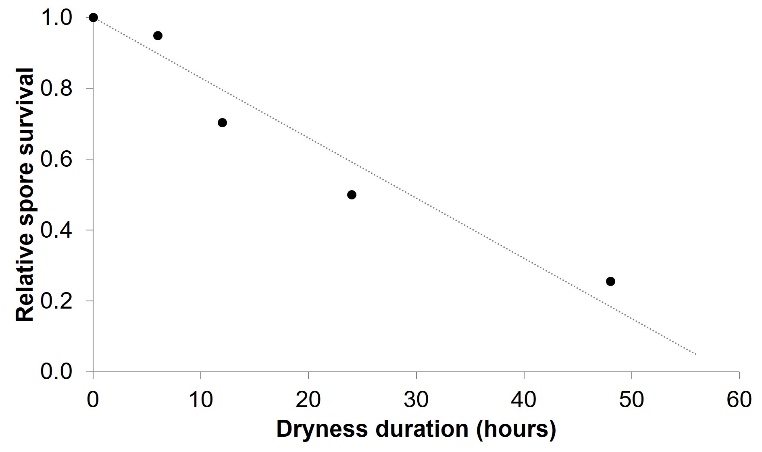


**Figure S3.** Dynamic of *Ascochyta rabiei* survival of ascospores and conidia. Dots show the data of Trapero-Casas and Kaiser [1], and the dotted line shows the fit of data with equation (3), with R^2^ = 0.937.

Equation (6, 7, 8)

Equations (6) and (7) of the model account for the hourly progress of incubation and latency, respectively. Equation (6) was developed by combining data from Basandrai *et al.* [3], Trapero-Casas and Kaiser [4], and Chauhan and Sinha [5]; equation (7) was obtained by fitting the data from Trapero-Casas and Kaiser [4]. Basandrai *et al.* [3] inoculated plants of susceptible cv. Pb 7 at different growth stages (seedling, post-seedling, vegetative, flowering, and pod formation) by spraying a suspension of conidia (5 x 10^4^ conidia per ml). Plants were incubated at 20°C and 100% RH for 1 week and were observed daily for symptom onset. For all growth stages, symptoms were recorded on the 3^rd^ day after inoculation. Chauhan and Sinha [5] determined the effect of temperature (from 10 to 30°C) on incubation time by inoculating plants of cv. T 87 (susceptible). Plants were grown for 45 days in a greenhouse before they were placed in moist chambers (100% RH) at different temperatures and inoculated with a conidial suspension (10^5^ conidia per ml). Plants were inspected daily for Ascochyta blight symptoms. Trapero-Casas and Kaiser [4] evaluated the effect of temperature on incubation and latency by placing chickpea seedlings that had been inoculated with conidia (5 x 10^5^ conidia per ml) and kept at 20°C in a saturated atmosphere for 48 h at 5, 10, 15, 20, 25, or 30°C. Plants were inspected daily for lesions and pycnidia. Incubation data retrieved from the three papers were pooled, and the average number of hours required from inoculation to lesion onset and pycnidia formation was calculated for each temperature. Time (hours) for lesion onset (independent variable Y) was fitted by the equation of Magarey *et al.* [6] in the form Y = f(T) / min, where f(T) = (T(i) - Tmin) / (Topt - Tmin) ((Tmax - T(i)) / (Tmax - Topt))^(Tmax - Topt) / (Topt - Tmin)^, as reported in equation (8). Min and cardinal temperatures were estimated by evaluating goodness-of-fit of a set of equations calculated by changing the min by 1 hour and the three cardinal temperatures in turn by 0.5°C. The best fit for incubation was obtained with min = 150, Tmin = 2°C, Topt = 21°C, and Tmax = 34°C, with R^2^=0.887 (Figure S4a). The equation of Magarey *et al.* [6] was also used to fit the data for latency [4], and the best fit was obtained with min = 168, Tmin = 2°C, Topt = 21°C, and Tmax = 34°C, with R^2^=0.945 (Figure S4b).

| 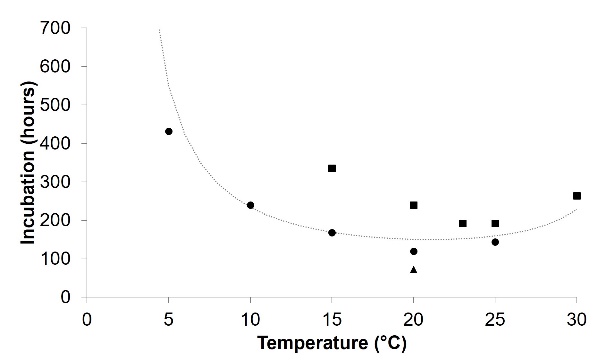 | 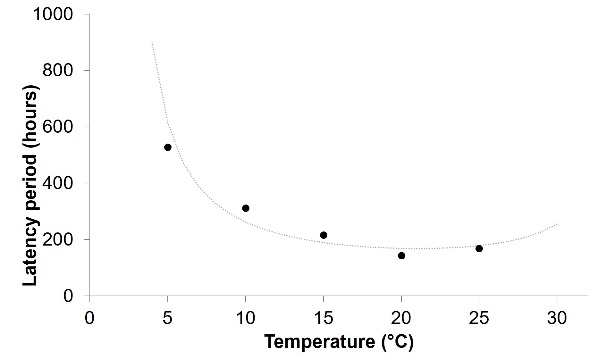 |
| --- | --- |
| (**a**) | (**b**) |

**Figure S4.** Effect of temperature on length of (**a**) incubation and (**b**) latency periods, expressed in hours after infection by *Ascochyta rabiei*. In (**a**) symbols show data of Basandrai *et al.* [3] (▲), Trapero-Casas and Kaiser [4] (●), and Chauhan and Sinha [5] (■); the dotted line shows the length of incubation as predicted by the equation of Magarey *et al.* [6] (6), with R^2^=0.887. In (**b**) dots show the average number of hours required for pycnidia formation as calculated using data of Trapero-Casas and Kaiser [4]; the dotted line shows the length of the incubation as predicted by the equation of Magarey *et al.* [6] (7), with R^2^=0.945.

Equation (9)

Equation (9) of the model was developed by using data from Trapero-Casas and Kaiser [1,4], Khan [7], and Weltzien and Kaack [8]. To determine the effect of temperature on infection, Trapero-Casas and Kaiser [1,4] sprayed 2-week-old chickpea seedlings cv. Bupree (susceptible) until runoff with a conidial suspension (5 x 10^5^ and 2 x 10^4^ conidia per ml in 1992 and 2007, respectively). Inoculated plants were incubated in moist chambers at 5, 10, 15, 20, 25, or 30°C. After 48 h, plants were removed from moist chambers, dried, and returned to the same temperature condition with a 14-h photoperiod, or were moved to a greenhouse at 18-26°C until lesion onset. Disease severity was recorded using a 0 (healthy plant)-to-9 scale (dead plant) scale. Khan [7] inoculated cv. Desavic (susceptible) plants with a conidial suspension (5 x 10^5^ conidia per ml) and kept them at 10, 15, 20, 25, or 30°C for 72 h. The plants were then dried, and were assessed for symptoms 14 days after the inoculation using the scale of Gowen *et al.* [9], which describes disease severity from 0-10% (no infection – small lesions) to 100% (plants completely dead). Weltzien and Kaack [8] inoculated chickpea plants at temperatures between 0 and 30°C in environmental chambers and kept them wet for 45 hours; after this period, plants were dried and moved to a greenhouse. Disease severity was recorded 14 days after inoculation using a scale with 9 grades based on the percentage of affected area. Data retrieved from these papers were rescaled from 0-to-1 to determine the relative infection severity. Relative infection severity (dependent variable Y) was regressed against temperature as the equivalent of temperature (Teq, independent variable X), which was calculated as for Equation (1) before. Cardinal temperatures were set based on Trapero-Casas and Kaiser [1], who found that conidia did not germinate at 0°C or 35°C; therefore, Tmin = 0°C and Tmax = 35°C.The best fit was obtained with a bete equation [2] in the following form: Y = (a X^b^ (1-X))^c^, with a = 5.200±0.386, b = 1.560±0.124, and c = 1.057±0.185, with R^2^ = 0.978 (Figure S5).


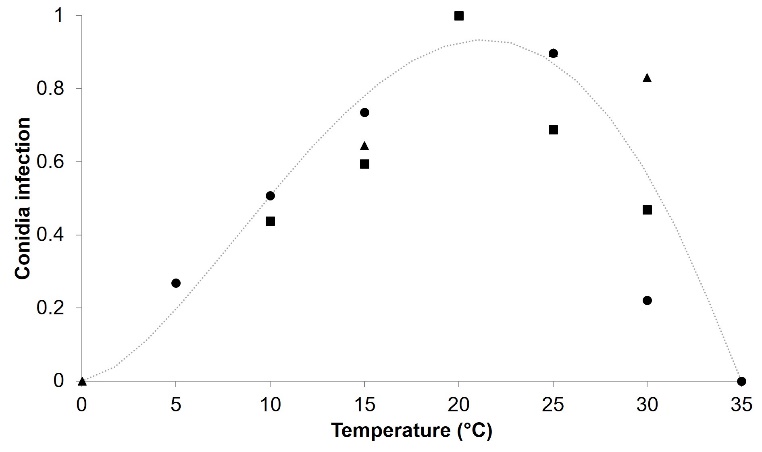


**Figure S5.** Relationship between relative infection severity of *Ascochyta rabiei* and temperature (°C). Symbols show the data of Trapero-Casas and Kaiser [1,4] (●), Khan [7] (▲), and Weltzien and Kaack [8] (■); the dotted line shows the fit of data using a bete equation (9) [2], with R^2^ = 0.978.

Equation (10)

Equation (10) was developed and parameterized by fitting the data of Trapero-Casas and Kaiser [1,4], Armstrong-Cho *et al.* [10], Khan [7], and Jhorar *et al.* [11]. Trapero-Casas and Kaiser [1,4] inoculated 2-week-old chickpea seedlings of cv. Burpee (susceptible) with a conidial suspension (5 x 10^5^ and 2 x 10^4^ conidia per ml in 1992 and 2007, respectively) until runoff. Plants were then incubated in moist chambers at 20°C in the dark. After 0, 6, 12, 24, or 48 hours, plants were removed from the chambers, dried, and incubated in a growth chamber at 20°C with a 14-h photoperiod and <50% RH for 7 days before being transferred to a greenhouse at 18-26°C. Disease severity was recorded using a 0-9 rating scale that described the percentage of disease from 0 (no infection) to 100% (dead plant). Armstrong-Cho ^et al.^ [10] inoculated seedlings of cv. UC27 (susceptible) with a suspension of 2 x 10^5^ conidia per ml and kept the plants in a mist chamber for 12, 24, 36, 48, 60, or 72 h before moving them to a dry environment; lesions were counted 2 weeks after inoculation. Khan [7] inoculated plants of cv. Desavic (susceptible) with a conidial suspension (5 x 10^5^ conidia per ml) at 20°C and for wet periods of 0, 6, 12, 24, 48, 72, or 96 hours; Ascochyta blight symptoms were assessed 14 days after the inoculation using the 0-9 scale of Gowen *et al.* [9]. Jhorar *et al.* [11] inoculated 15-day-old chickpea plants cv. L-550 (susceptible) with a conidial suspension (10^6^ conidia per ml) at 20°C for wet periods of 0, 2, 3, 4, 6, 12, 18, or 24 hours. At the end of each wet period, plants were dried and moved to a growth chamber until symptoms appeared and disease severity was assessed. Data from all these experiments, which were expressed as % disease severity or number of lesions, were rescaled relative to the maximum of each experiment to obtain rescaled values ranging from 0 to 1. The resulting relative infection severity (independent variable Y) was regressed against the duration of wet period. The best fit was obtained using a monomolecular function in the following form: Y = 1 - b exp(- a X), where a = 0.034±0.004 and b = 1.000±0.042, with R^2^ = 0.944 (Figure S6). The first-order derivative of this equation was used as equation (11) of the model.


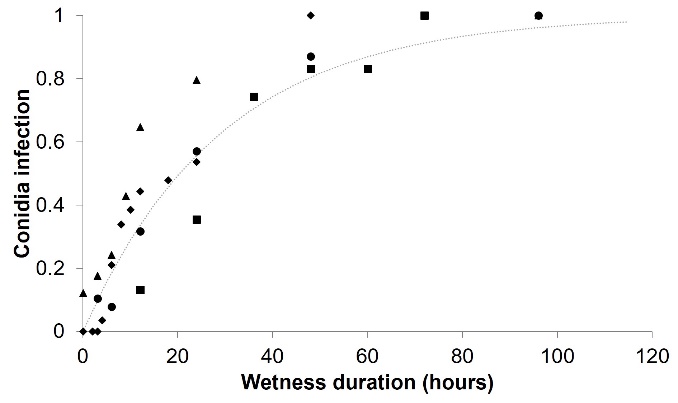


**Figure S6.** Relationship between relative infection severity of *Ascochyta rabiei* and wetness duration (hours). Symbols show the data of Trapero-Casas and Kaiser [1,4] (●), Armstrong-Cho *et al.* [10] (■), Khan [7] (▲), and Jhorar *et al.* [11] (♦); the dotted line shows the fit of data with equation (10), with R^2^ = 0.944.

References

1. Trapero-Casas, A.; Kaiser, W.J. Differences between ascospores and conidia of *Didymella rabiei* in spore germination and infection of chickpea. *Phytopathology* **2007**, 97, 1600–1607.
2. Analytis, S. On the relation between biological development and temperature of some plant pathogenic fungi. *Phytopathology* **1977**, 90, 64–76.
3. Basandrai, A.K.; Basandrai, D.; Pande, S.; Sharma, M.; Thakur, S.K.; Thakur, H.L. Development of Ascochyta blight (*Ascochyta Ascochyta*) in chickpea as affected by host resistance and plant age. *Eur. J. Plant Pathol.* **2007**, 119, 77–86.
4. Trapero-Casas, A.; Kaiser, W.J. Influence of temperature, wetness period, plant age and inoculum concentration on infection and development of Ascochyta blight of chickpea. *Phytopathology* **1992**, 82, 589–596.
5. Chauhan, R. K. S.; Sinha, S. Effect of varying temperature, humidity and light during incubation in relation to disease development in blight of gram (*Cicer arietinum*) caused by *Ascochyta rabiei*. *Proc. Indian National Sci. Acad.* **1973**, 37, 473–482.
6. Magarey, R.D.; Sutton, T.B.; Thayer, C.L. A simple generic infection model for foliar fungal plant pathogens. *Phytopathology* **2005**, 95, 92–100.
7. Khan, A. Epidemiology of Ascochyta blight of chickpea in Australia. Doctoral dissertation, University of Adelaide, South Australia, June 1999.
8. Weltzien, H. C., Kaack, H.J. Epidemiological aspects of chickpea Ascochyta blight. In *Ascochyta blight and winter sowing of chickpeas*, Proceedings of Workshop on Ascochyta Blight and Winter Sowing of Chickpeas, Aleppo, Syria, 4–7 May 1981; Saxena, M.C., Singh, K.B., Eds; pp. 35–44.
9. Gowen, S.R.; Orton, M.; Thurley, B.; White, A. Variation in pathogenicity of *Ascochyta rabiei* on chickpeas. *Trop. Pest Manag.* **1989**, 35, 180–186.
10. Armstrong-Cho, C.; Gossen, B.D.; Chongo, G. Impact of continuous or interrupted leaf wetness on infection of chickpea by *Ascochyta rabiei*. *Can. J. Plant Pathol.* **2004**, 26, 134–141.
11. Jhorar, O.P.; Butler, D.R.; Mathauda, S.S. Effects of leaf wetness duration, relative humidity, light and dark on infection and sporulation by *Didymella rabiei* on chickpea. *Plant Pathol.* **1998**, 47, 586–594.
