# Supplementary figures and images for: A Mechanistic Weather-Driven Model for Ascochyta rabiei Infection and Disease Development in Chickpea
Source: Plants (Basel). 2021 Mar 1;10(3):464. doi: 10.3390/plants10030464 (PMC8000037; doi:10.3390/plants10030464)

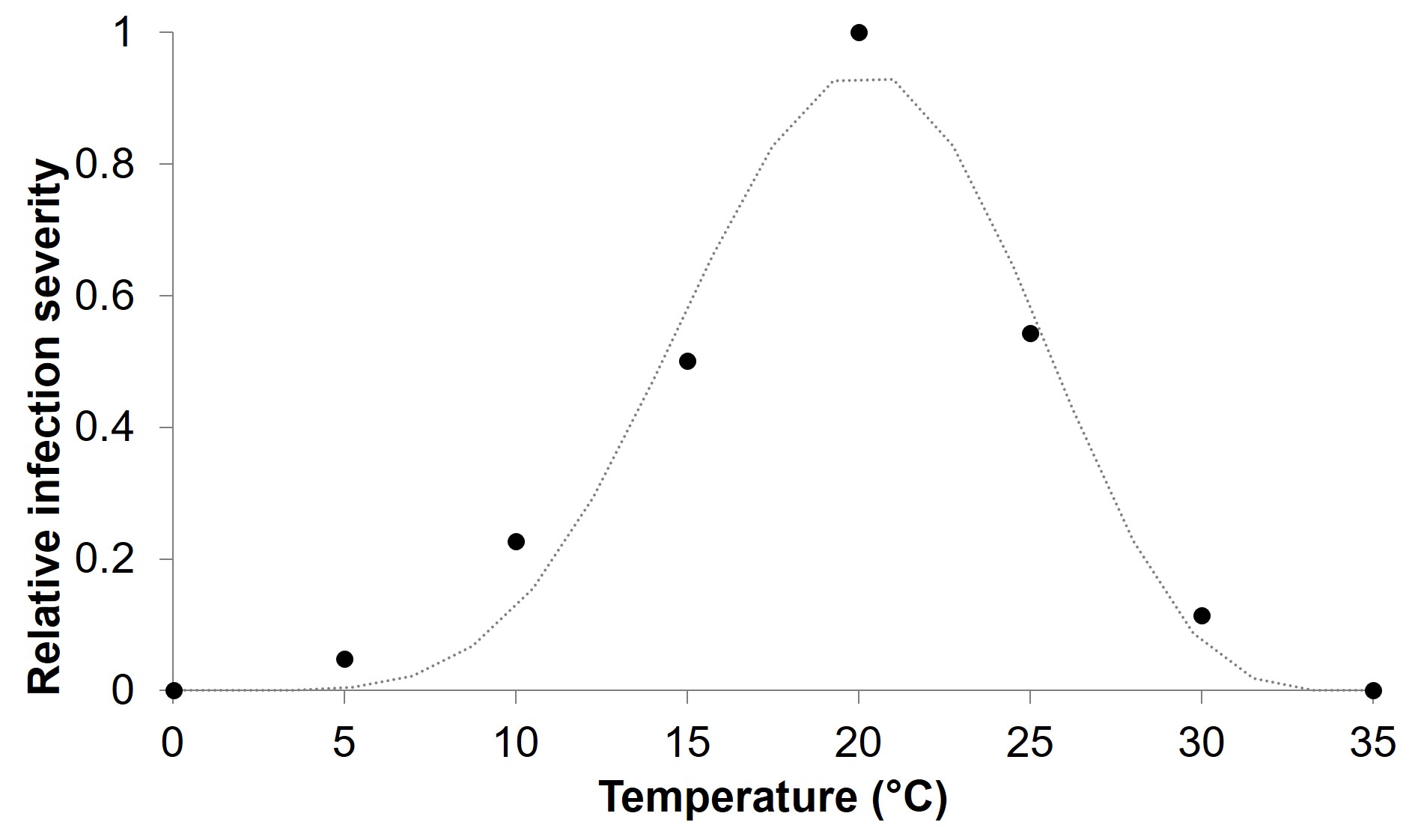

Supplement: Supplementary file 1 [file plants-10-00464-s001.zip › Supplementary/Figures supplementary/Figure S1.jpg]

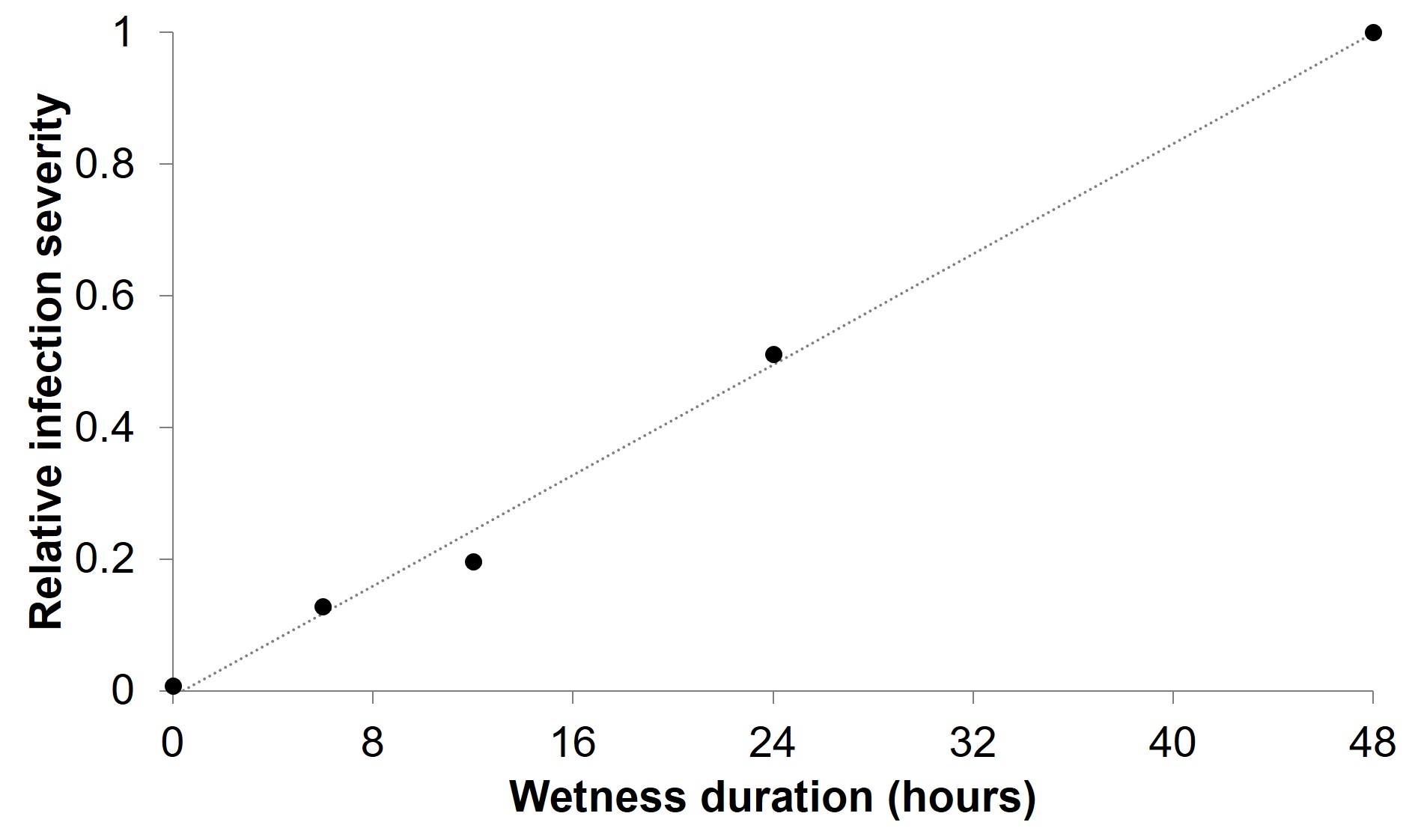

Supplement: Supplementary file 1 [file plants-10-00464-s001.zip › Supplementary/Figures supplementary/Figure S2.jpg]

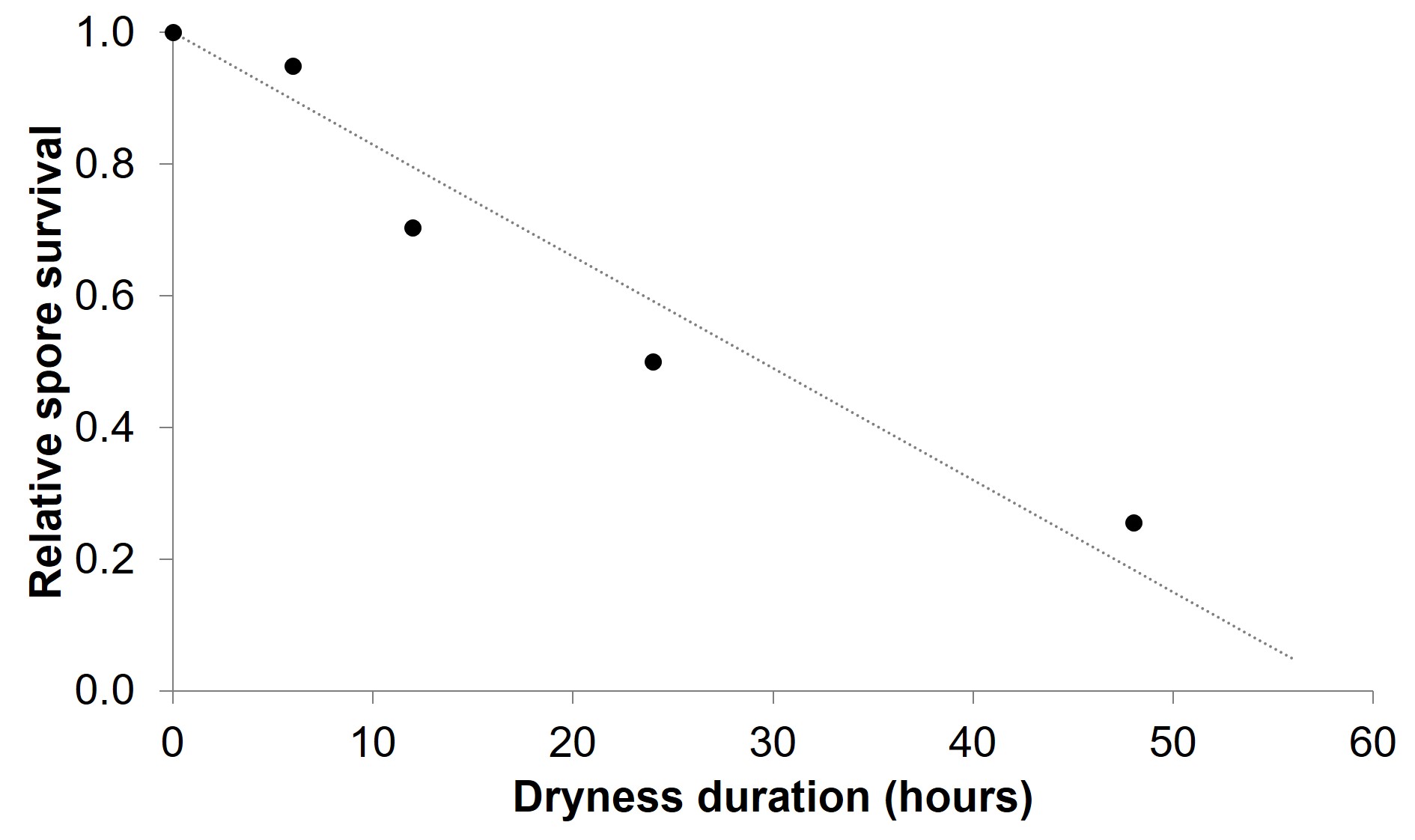

Supplement: Supplementary file 1 [file plants-10-00464-s001.zip › Supplementary/Figures supplementary/Figure S3.jpg]

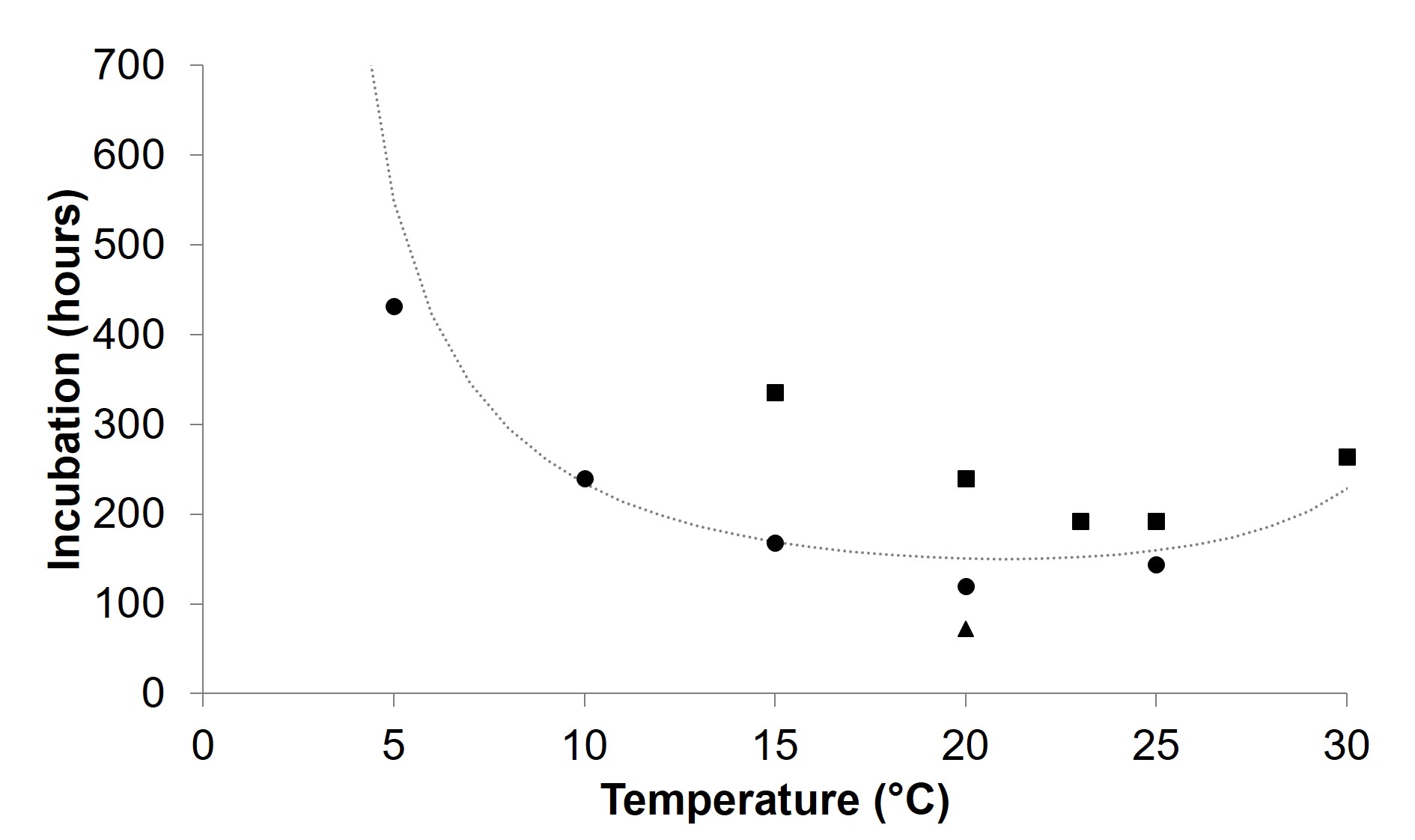

Supplement: Supplementary file 1 [file plants-10-00464-s001.zip › Supplementary/Figures supplementary/Figure S4a.jpg]

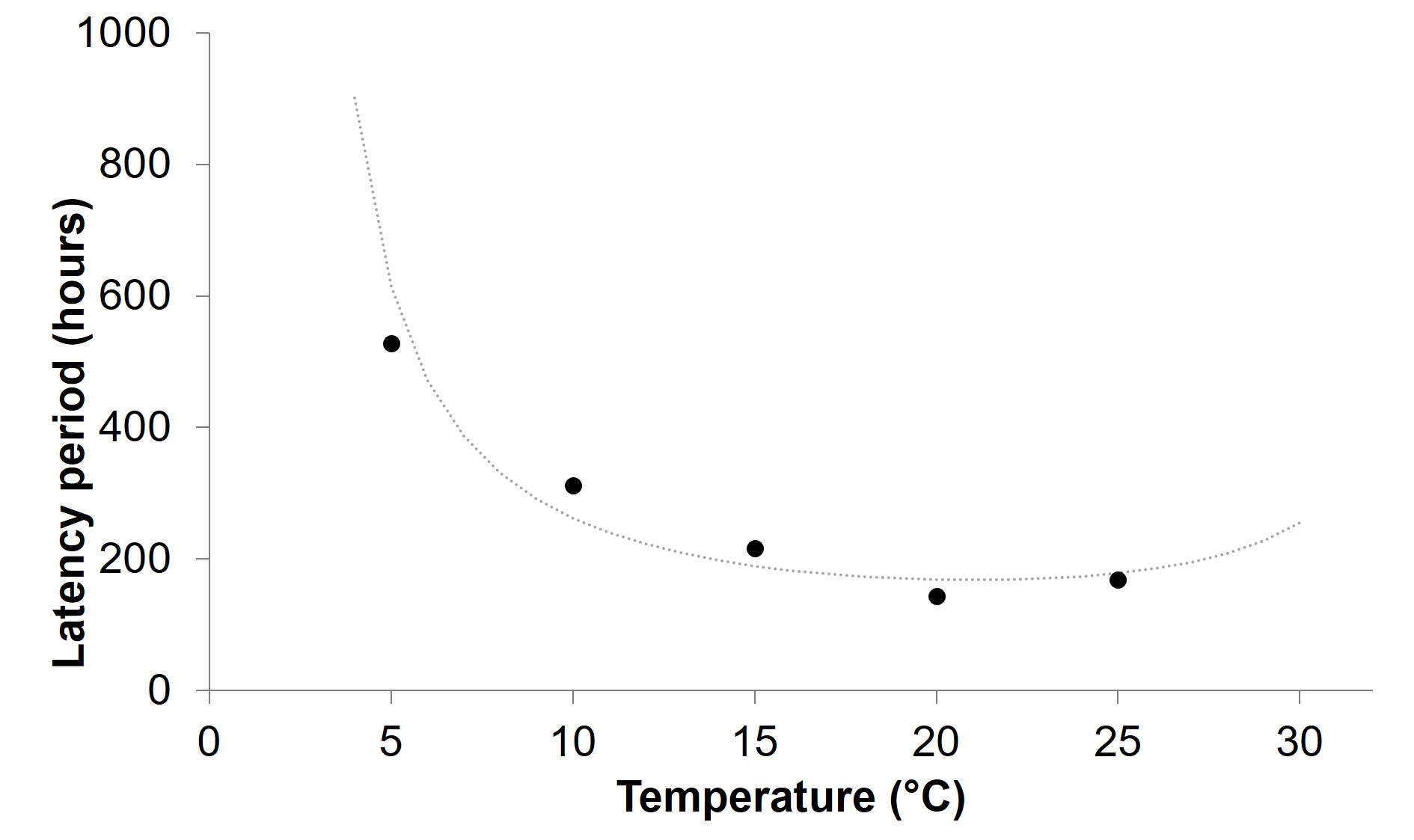

Supplement: Supplementary file 1 [file plants-10-00464-s001.zip › Supplementary/Figures supplementary/Figure S4b.jpg]

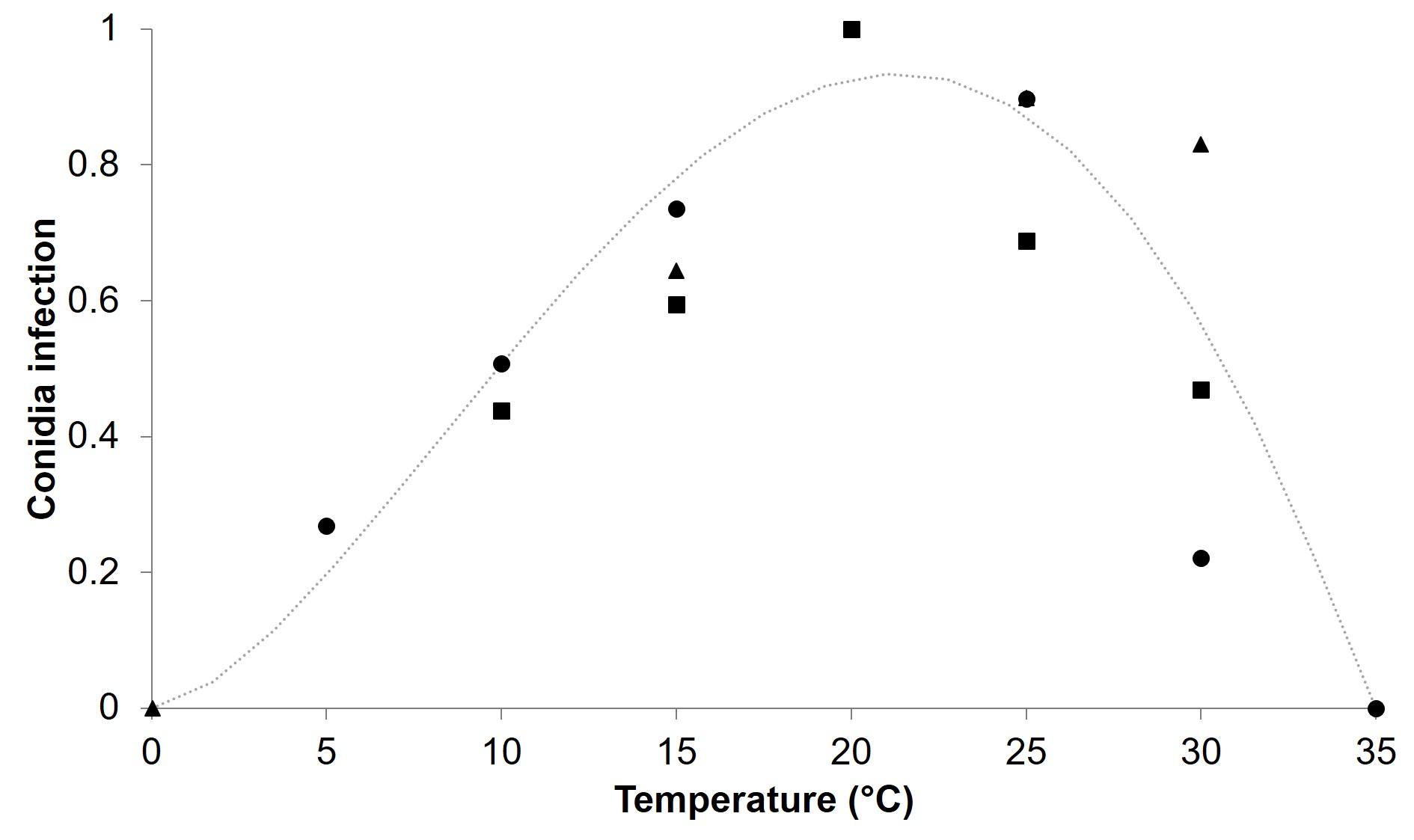

Supplement: Supplementary file 1 [file plants-10-00464-s001.zip › Supplementary/Figures supplementary/Figure S5.jpg]

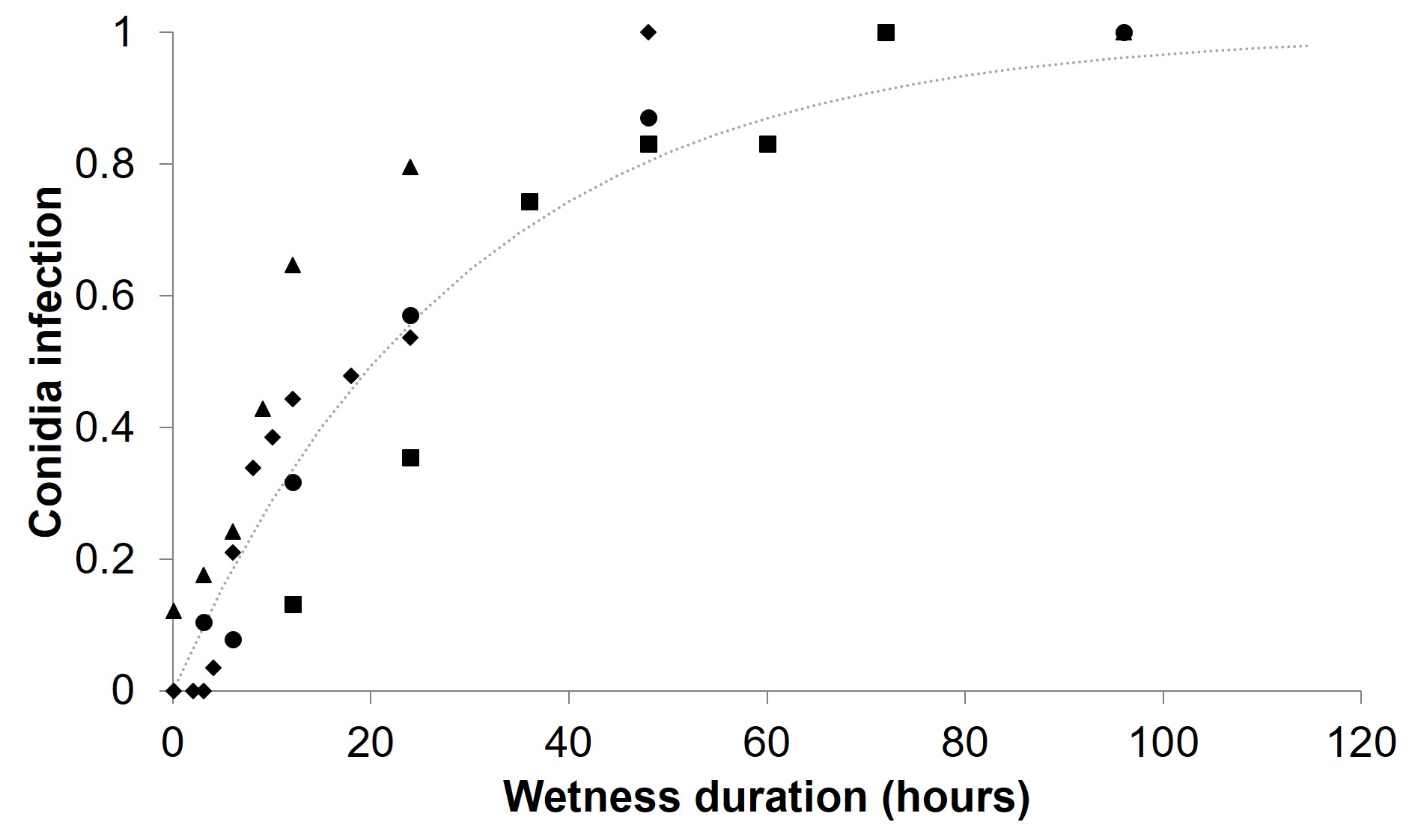

Supplement: Supplementary file 1 [file plants-10-00464-s001.zip › Supplementary/Figures supplementary/Figure S6.jpg]
